# Music-Evoked Emotions—Current Studies

**DOI:** 10.3389/fnins.2017.00600

**Published:** 2017-11-24

**Authors:** Hans-Eckhardt Schaefer

**Affiliations:** ^1^Tübingen University, Institute of Musicology, Tübingen, Germany; ^2^Institute of Functional Matter and Quantum Technology, Stuttgart University, Stuttgart, Germany

**Keywords:** emotions, music, music therapy, EEG, fMRI

## Abstract

The present study is focused on a review of the current state of investigating music-evoked emotions experimentally, theoretically and with respect to their therapeutic potentials. After a concise historical overview and a schematic of the hearing mechanisms, experimental studies on music listeners and on music performers are discussed, starting with the presentation of characteristic musical stimuli and the basic features of tomographic imaging of emotional activation in the brain, such as functional magnetic resonance imaging (fMRI) and positron emission tomography (PET), which offer high spatial resolution in the millimeter range. The progress in correlating activation imaging in the brain to the psychological understanding of music-evoked emotion is demonstrated and some prospects for future research are outlined. Research in psychoneuroendocrinology and molecular markers is reviewed in the context of music-evoked emotions and the results indicate that the research in this area should be intensified. An assessment of studies involving measuring techniques with high temporal resolution down to the 10 ms range, as, e.g., electroencephalography (EEG), event-related brain potentials (ERP), magnetoencephalography (MEG), skin conductance response (SCR), finger temperature, and goose bump development (piloerection) can yield information on the dynamics and kinetics of emotion. Genetic investigations reviewed suggest the heredity transmission of a predilection for music. Theoretical approaches to musical emotion are directed to a unified model for experimental neurological evidence and aesthetic judgment. Finally, the reports on musical therapy are briefly outlined. The study concludes with an outlook on emerging technologies and future research fields.

## Introduction

Basic discussions of music center about questions such as: What actually is music? How can we understand music? What is the effect of music on human beings? Music is described as multidimensional and researchers have categorized it by its arousal properties (relaxing/calming vs. stimulating), emotional quality (happy, sad, peaceful), and structural features (as, e.g., tempo, tonality, pitch range, timbre, rhythmic structure) (Chanda and Levitin, 2013). One can ask the question how to recognize and describe the concretely beautiful in music. Efforts have been undertaken to answer this question (Eggebrecht, 1991), e.g., by discussing the beauty of the opening theme of the second movement of Mozart's piano concerto in d minor (KV 466). In this formal attempt to transform music into a descriptive language, particular sequences of tones and rhythmical structures have been tentatively ascribed to notions such as “flattering” or “steady-firm” (Eggebrecht, 1991). From the viewpoint of a composer, Mozart himself obviously was aware of the attractiveness of this beauty-component in music, stating that his compositions should be “…angenehm für die Ohren…” of the audience “…natürlich ohne in das Leere zu fallen…” (…pleasing for the ear… (of the audience) …naturally without falling into the shallow…) (see Eggebrecht, 1991). In modern and contemporary music, however, formal attempts of understanding are useless because form and self-containedness are missing (Zender, 2014). Thus, in atonality and in the emancipation of noise, a tonal center is absent, by simultaneous appearance of different rhythmic sequences the regular meter is demolished, and in aleatory music the linear order of musical events is left open.

A few earlier comments on the understanding of the interplay between music and man may be quoted here: “…there is little to be gained by investigation of emotion in music when we have little idea about the true fundamental qualities of emotion” (Meyer, 1956). “…music is so individual that attempts to provide a systematic explanation of the interaction might well be ultimately fruitless—there may be no systematic explanation of what happens when individuals interact with music” (Waterman, 1996). “Die Qualitäten und die Inhalte ihrer (der Komponisten) Musik zu beschreiben ist unmöglich. Eben deshalb werden sie in Klang gefasst, weil sie sonst nicht erfahrbar sind” (To describe the qualities and content of their (of the composers) music is impossible. Exactly for this reason they are expressed in musical sound, otherwise they are not communicable) (Maurer, 2014). Some historical comments on music-evoked emotions are compiled in section Historical Comments on the Impact of Music on People of this study.

The advent of brain-imaging technology with high spatial resolution (see principles section Experimental Procedures for Tomographic Imaging of Emotion in the Brain) gave new impact to interdisciplinary experimental research in the field of music-evoked emotions from the physiological and molecular point of view. With the broader availability of magnetic resonance imaging (MRI, first demonstrated in 1973; Lauterbur, 1973) and positron emission tomography (PET, first demonstrated 1975; Ter-Pogossian, 1975) since about two decades for studying both music listeners and performing musicians, a wealth of music-evoked brain activation data has been accomplished which is discussed in section Experimental Results of Functional (tomographic) Brain Imaging (fMRI, PET) together with psychoendocrinological and molecular markers. Due to the refinement of the more phenomenological measuring techniques, such as electroencephalography (EEG) and magnetoencephalography [MEG, section Electro- and Magnetoencephalography (EEG, MEG)], skin conductance response and finger temperature measurements (section Skin Conductance Response (SCR) and Finger Temperature) as well as goose bump development (section Goose Bumps—Piloerection), emotions can be measured with high temporal resolution. Genetic studies of musical heredity are reported in section Is There a Biological Background for the Attractiveness of Music?—Genomic Studies and recent theoretical approaches of musical emotions in section Towards a Theory of Musical Emotions. Some therapeutic issues of music are discussed in section Musical Therapy for Psychiatric or Neurologic Impairments and Deficiencies in Music Perception prior to the remarks concluding this study with an outlook. A brief outline of the psychological discussion of music-evoked emotion is given in the online Supplementary Material section.

## Historical comments on the impact of music on people

The effects of music on man have been considered phenomenologically from antiquity to the nineteenth century mainly from the medical point of view according to Kümmel (1977) which will be preferentially referred to in the brief historical comments of the present section.

The only biblical example of a healing power of music refers to King Saul (~1,000 BC) who was tormented by an evil spirit and relief came to him when David played the lyre (1. Sam. 16, 14-23). In Antiquity, Pythagoras (~570-507 BC) was said to substantially affect the souls of people by diatonic, chromatic, or enharmonic tunes (see Kümmel, 1977). Platon (428-348 BC) in his *Timaios* suggested for the structure of the soul the same proportions of the musical intervals which are characteristic for the trajectories of the celestial bodies (see Kümmel, 1977). This concept of a numeral order of music and its effect on man was transferred to the Middle Ages, e.g., by Boethius (480-525). The Greek physician Asklepiades (124-60 BC) was said to have used music as a remedy for mental illness where the application of the Phrygian mode was considered to be particularly adequate for brightening up depressive patients. Boethius emphasized that music has to be correlated to the category of “moralitas” because of its strong effect on individuals. In his treatise *De institutione musica* he stated that “…music is so naturally united with us that we cannot be free from it even if we so desired….” Since the ninth century, music took a strong position in the medicine of the Arabic world and the musician was an assisting professional of the physician. According to Arabic physicians, music for therapeutic purposes should be “pleasant,” “dulcet,” “mild,” “lovely,” “charming,” and in the course of the assimilation of the Arabic medicine, the Latin West took over the medical application of music. Johannes Tinctoris (1435-1511) listed 20 effects of music, such as, e.g., that music banishes unhappiness, contributes to a cheerful mood, and cures diseases. In addition, music was supposed to delay aging processes. Agrippa von Nettesheim (1486-1535) was convinced that music can maintain physical health and emboss a moral behavior. He discusses in his treatise *De occulta philosophia* (Agrippa von Nettesheim, 1992) the powerful and prodigious effects of music. From his list of 20 different musical effects—adapted to the sequence of effects established by Johannes Tinctoris (1435-1511) (Schipperges, 2003) a brief selection should be presented here:

(1) Musica Deum delectat
(7) Musica tristitiam repellit
(13) Musica homines laetificat
(14) Musica aegrotos sanat
(17) Musica amorem allicit etc.

These effects could be translated into nowadays notions as religiosity (1), depression (7), joy (13), therapy (14), and sexuality (17).

Agrippa points out the alluring effects of music on unreasoning beasts: “…ipsas quoque bestias, serpentes, volucres, delphines, ad auditum suae modulationis provocat…magna vis est musica” (It stirs the very beasts, even serpents, birds and dolphins, to want to hear its melody…great is the power of music).

The physician of Arnstadt, Johann Wittich (1537-1598) summarized the requirement for good health concisely: “Das Hertz zu erfrewen/und allen Unmuht zu wenden/haben sonderliche große Krafft diese fünff Stück (To rejoice the heart/ and reverse all discontent/five things have particularly great power):

1. Gottes Wort (The word of God).
2. Ein gutes Gewissen (A clear conscience).
3. Die Musica (Music).
4. Ein guter Wein (good wine).
5. Ein vernünftig Weib (A sensible wife).”

**Figure 1:**
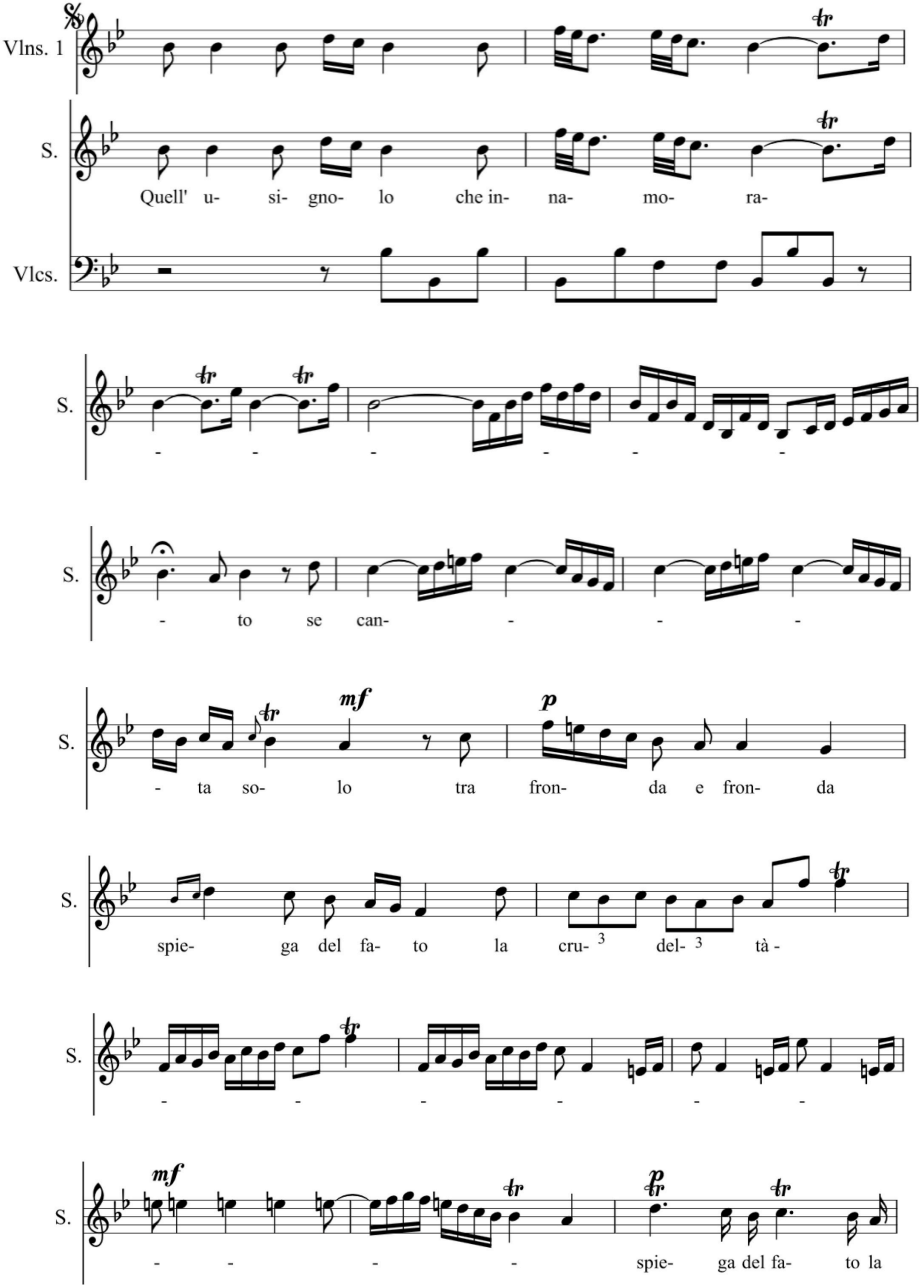
Extract from the aria *Quell' usignolo* of Geminiano Giacomelli's (1692-1740) opera *Merope* (1734) sung by Carlo Broschi Farinelli (1705-1782) for Philipp V (1683-1746), king of Spain (Haböck, 1923). Reprinted with permission from Haböck (1923) © 1923 Universal Edition.

René Descartes (1596-1650) formulated a fairly detailed view of the effects of music: The same music which stimulates some people to dancing may move others to tears. This exclusively depends on the thoughts which are aroused in our memory. In the medical encyclopedia of Bartolomeo Castelli of 1682 it is stated that music is efficient for both the curing of diseases and for maintaining health. A famous historical example for a positive impact of music on mental disorders is the Spanish King Philipp V (1683-1746) who—due to his severe depressions—stopped signing official documents and got up from his bed only briefly and only by night. In 1737, his wife Elisabeth Farnese (1692-1766, by the way a descendant of Pope Paul III and Emperor Karl V) appointed the famous Italian castrato singer Carlo Broschi Farinelli (1705-1782) to Madrid. Over 10 years, Farinelli performed every night (in total 3,600 times) four arias in order to banish the black melancholia from the kings mind until the king himself “…die Musik lernet…” (…learns music…) (see Kümmel, 1977). With his singing, Farinelli succeeded in agitating the king to partial fulfillment of his governmental duties and an occasional appearance in the governmental council. The king's favorite aria was *Quell' usignolo* with a difficult coloratura part (see Figure 1) of Geminiano Giacomelli's (1692-1740) opera *Merope* (1734).

The widely known *Goldberg Variationen* composed by J. S. Bach in 1740 may be considered, as reported by Bach biographer J. N. Forkel (1749-1818), as therapeutic music. H. C. von Keyserlingk, a Russian diplomat, asked Bach for “…einige Clavierstücke für seinen Adlatus Johann Gottlieb Goldberg,…die so sanften und etwas munteren Charakters wären, daß er dadurch in seinen schlaflosen Nächten ein wenig aufgeheitert werden könnte…” (… a number of clavier pieces for his personal assistant J. G. Goldberg…which should be of such gentle and happy character that he be somewhat cheered in his sleepless nights…). Bach chose a variations composition because of the unchanged basic harmony, although he initially had regarded a piece of this technique as a thankless task (see Kümmel, 1977).

In 1745 the medicine professor E. A. Nicolai (1722-1802) of Jena University started to report on more physical observations: “… wenn man Musik höre richten sich die Haare …in die Höhe, das Blut bewegt sich von aussen nach innen, die äusseren Teile fangen an kalt zu werden, das Herz klopft geschwinder und man hohlt etwas langsamer und tiefer Athem” (…when one hears music the hair stands on end (see section Goose Bumps—Piloerection), the blood is withdrawn from the surface, the outer parts begin to cool, the heart beats faster, and one breathes somewhat slower and more deeply). The French *Encyclopédie* of 1765 listed the diseases for which music was to be employed therapeutically: Pathological anxieties, the bluster of mental patients, gout pain, melancholia, epilepsy, fever, and plague. The physician and composer F. A. Weber (1753-1806) of Heilbronn, Germany assessed in 1802 the health effects of music more reluctantly: “Nur in Übeln aus der Klasse der Nervenkrankheiten läßt sich von…der Musik etwas Gedeihliches erhoffen. Vollständige Impotenz ist durch Musik nicht heilbar…Allein als Erwärmungsmittel erkaltender ehelicher Zärtlichkeit mag Musik vieles leisten” (Only in afflictions of the class of nervous diseases can …something profitable be expected from music. Complete impotence is not curable by music. …But as a means of rekindling marital tenderness music may achieve considerable results). The French psychiatrist J. E. D. Esquirol (1772-1840, see Charland, 2010) started to perform numerous experiments with the application of music to single patients or to groups. He, however, stated that the effect of music was transient and disappeared when the music ended. This change of thinking is also visible in the essay by Eduard Hanslick (1825-1904) *Vom musikalisch Schönen* (1854): “Die körperliche Wirkung der Musik ist weder an sich so stark, noch so sicher, noch von psychischen und ästhetischen Voraussetzungen so unabhängig, noch endlich so willkürlich behandelbar, dass sie als wirkliches Heilmittel in Betracht kommen könnte” (The physical effect of music is as such neither sufficiently strong, consistent, free from psychic and aesthetic preconditions nor freely usable as to allow its use as a real medical treatment).

With the rise of the experimental techniques of natural sciences in the medicine of the late nineteenth century, the views, patterns, and notions as determined by musical harmony began to take a backseat. It should be mentioned here that skepticism with regard to the effects of music arose in early times. In the third century Quintus Serenus declared the banishing of fever by means of vocals as pure superstition. In 1650 Athanasius Kircher wrote: “Denn dass durch (die Musik) ein Schwindsüchtiger, ein Epileptiker oder ein Gicht-Fall…geheilt werden können, halte ich für unmöglich.” (For I hold it for impossible that a consumptive, an epileptic or a gout sufferer …could be cured by music).

## The mechanisms of hearing

**Figure 2:**
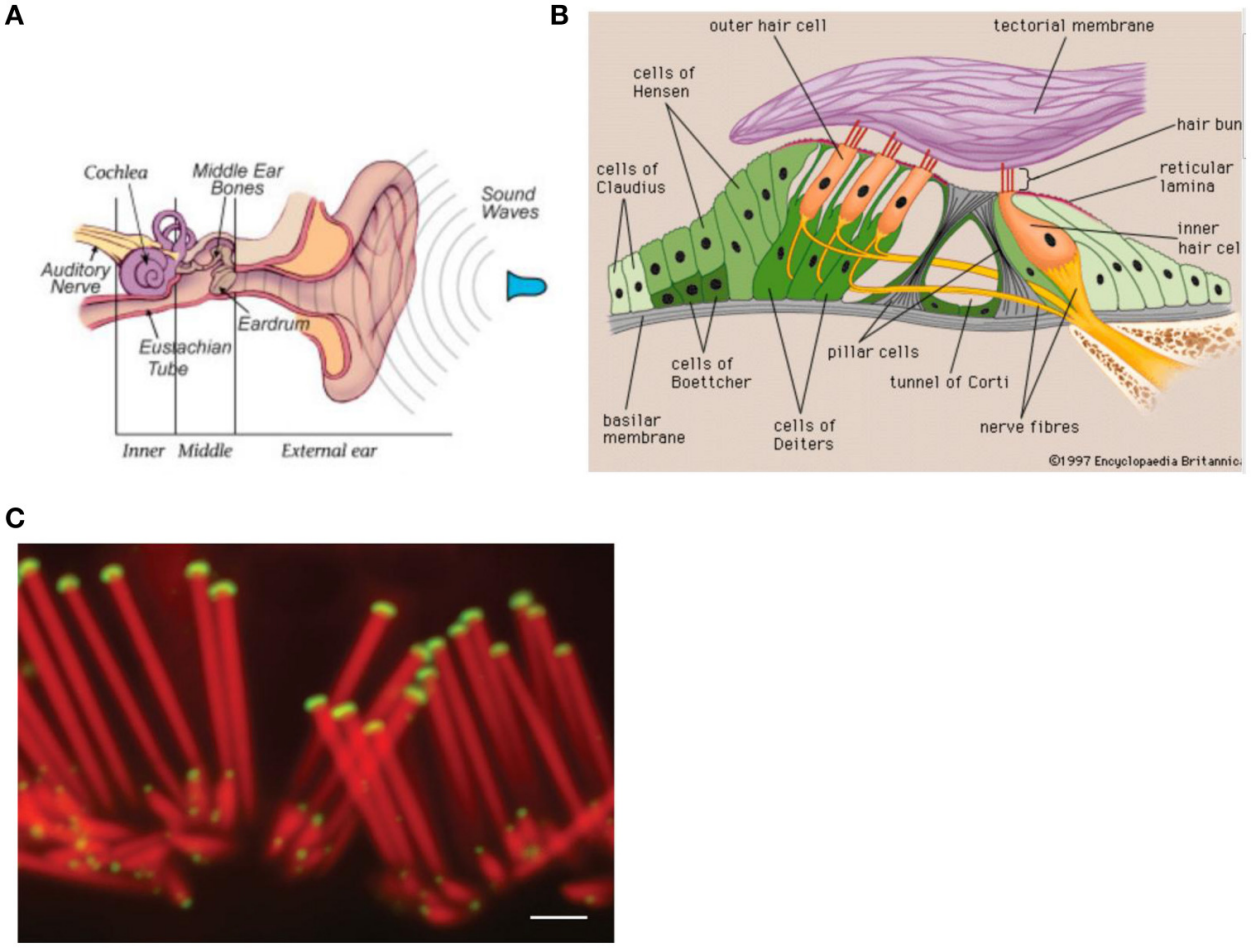
Anatomy of the ear. Reprinted with permission from William E. Brownell © 2016. **(B)** Components of the inner ear. Reprinted with permission from © 2016 Encyclopedia Britannica. **(C)** Confocal micrographs of rat auditory hair cells. Scale bar: 1 μm. The protein myosin XVa is localized to the stereocilia tips (Rzadzinska et al., 2004). Reprinted with permission from Rzadzinska et al. (2004) © 2016 Bechara Kachar.

Sound waves are detected by the ear and converted into neural signals which are sent to the brain. The ear has three divisions: The external, the middle, and the inner ear (see Figure 2A). The sound waves vibrate the ear drum which is connected to the ear bones (malleus, incus, and stapes) in the middle ear that mechanically carry the sound waves to the frequency-sensitive cochlea (35 mm in length, Figure 2B) with the basilar membrane in the inner ear. Here, making use of the cochlear hair cells (organ of Corti), the sound waves are converted into neural signals which are passed to the brain via the auditory nerve (Zenner, 1994). For each frequency, there is a region of maximum stimulation, or resonance region, on the basilar membrane. The spatial position x along the basilar membrane of the responding hair cells and the associated neurons determine the primary sensation of the pitch. A change in frequency of a pure tone causes a shift of the position of the activated region. This shift is then interpreted as a change in pitch (see Roederer, 2008) effect and laser studies allowed for a precise measurement of the movement of the basilar membrane (see Roederer, 2008).

The cochlear hair cells assist in relaying sound to the brain. The about 20,000 hair cells in the human ear are covered by stereocilia (see Figure 2C), giving them a hairy look. The stereocilia of the hair cell, which is sitting on the basilar membrane, are the primary structures used in sound transduction. With acoustic stimulation, the stereocilia bend which causes a signal that goes to the auditory nerve (see Figure 2A) and eventually to the auditory cortex allowing sound to be processed by the brain.

At loudest sound the bending amplitude of the stereocilia is about their diameter of 200 nm (a nanometer nm is a millionth of a mm) and at auditory threshold the movement is about 1 nm or, in the order of the diameter of small molecules (Fettiplace and Hackney, 2006), i.e., close to the thermal equilibrium fluctuations of the Brownian motion in the surrounding lymphatic liquid (Roederer, 2008).

The bending of the stereocilia initiates an uptake of potassium ions (K^+^) which in turn opens voltage-dependent calcium ion (Ca^+^) channels. This causes neurotransmitter release at the basal end of the hair cell, eliciting an action potential in the dendrites of the auditory nerve (Gray, 0000).

The action speed of the hair cells is incredibly high to satisfy the amazing demands for speed in the auditory system. Signal detection and amplification must be preferentially handled by processes occurring within one hair cell. The acoustic apparatus cannot afford the “leisurely pace” of the nervous system that works on a time scale of several milliseconds or more.

## Specific experimental techniques for studying musical emotion and discussion of the results

### Emotionally relevant musical stimuli

Emotional relevance of music is ascribed, e.g., to enharmonic interchange, starting of a singing voice, the climax of a crescendo, a downward quint, or in general a musically unexpected material (Spitzer, 2003, 2014). Four musical parameters for the activation of emotions appear to be particularly prominent in the literature (Kreutz et al., 2012): musical tempo, consonance, timbre, and loudness. Musical tempo could influence cardiovascular dynamics. The category of consonance could be associated with activation in the paralimbic and cortical brain areas (Blood and Zatorre, 2001) whereas dissonances containing partials with non-integer (irrational) frequency ratios may give rise to a sensation of roughness. The loudness or the physical sound pressure seems to be of relevance to psychoneuroendocrinological responses to music. Thus, crescendo leads to specific modulation of cardiovascular activity (see Kreutz et al., 2012), such as musical expectancy and tension (Koelsch, 2014). Musical sounds are often structured in time, space, and intensity. Several structural factors in music give rise to musical tension: consonance or dissonance, loudness, pitch, and timber can modulate tension. Sensory consonance and dissonance are already represented in the brainstem (Tramo et al., 2001) and modulate activity in the amygdala.

**Figure 3:**
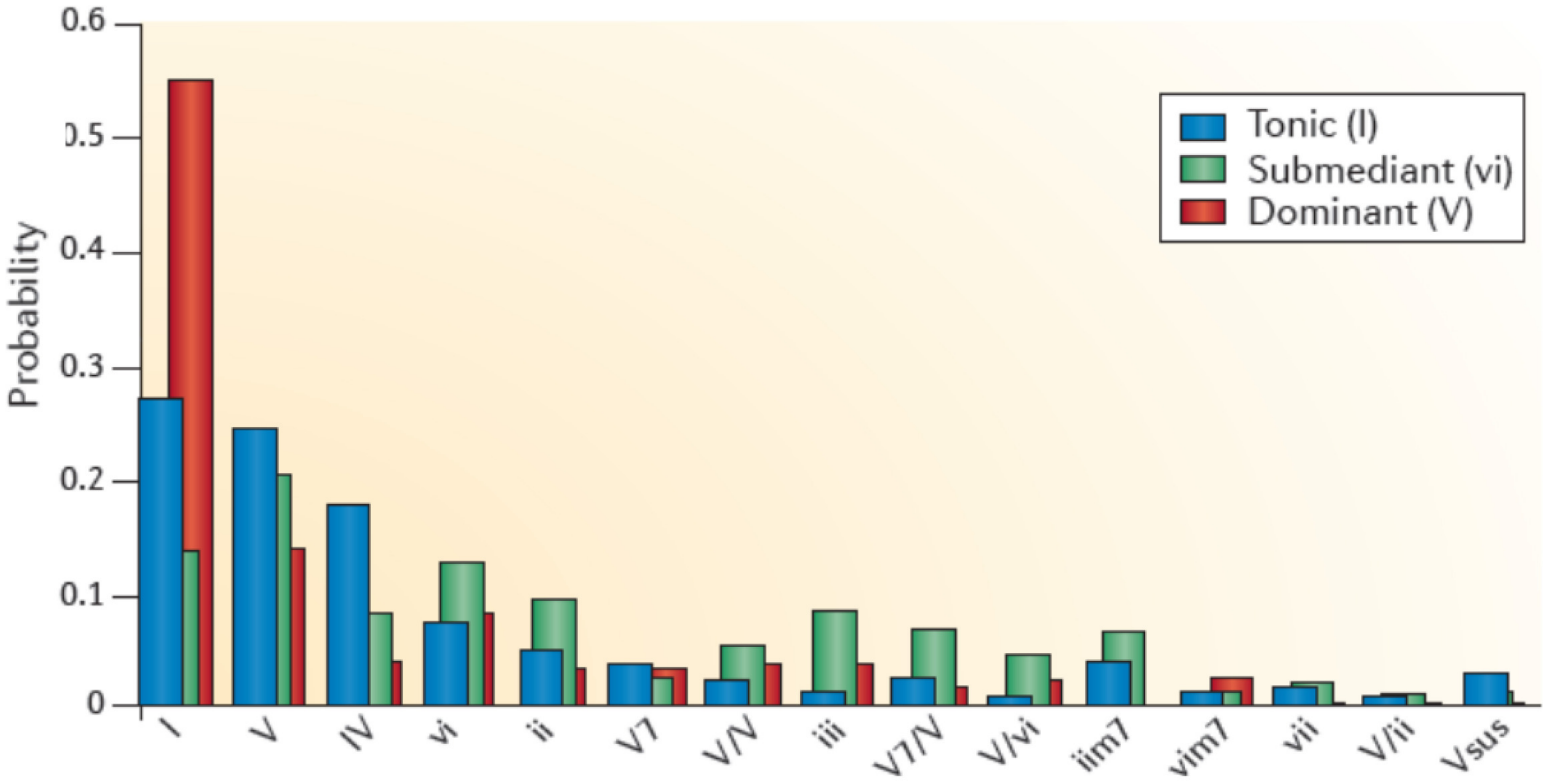
This graph shows the context-dependent bigram probabilities for the corpus of Bach chorales. Blue bars show probabilities of chord functions following the tonic (I), green bars following the submediant (vi), and red bars following a dominant (V). The probability for, e.g., a tonic (I) following a dominant (V) is high, the entropy is low (Koelsch, 2014). Reprinted with permission from Koelsch (2014) © 2014 Nature Publishing Group.

The stability of a musical structure also contributes to tension, such as a stable beat or its perturbation (for example, by an accelerando or a ritardando, syncopations, off-beat phrasings, etc.) (Koelsch, 2014). The stability of a tonal structure in tonal music also contributes to tension. Moving away from the tonal center creates tension and returning to it evokes relaxation. Figure 3 illustrates how the entropy of the frequency of the occurrence of tones and chords determines the stability of a tonal structure and thus the ease, or the difficulty, of establishing a tonal center. Additionally, the extent of a structural context contributes to tension. Figure 3 shows the probabilities of certain chords following other chords in Bach chorales. The red bars indicate that after a dominant the next chord is most likely to be a tonic. The uncertainty of the predictions for the next chord (and thus the entropy of the probability distribution for the next chord) is low during the dominant, intermediate during the tonic, and relatively high during the submediant. Progressive tones and harmonies thus create an entropic flux that gives rise to constantly changing (un)certainties of predictions. The increasing complexity of regulations, and thus the increase of entropic flux, requires an increasing amount of knowledge about the musical regularities to make precise predictions about upcoming events. Tensions emerge from the suspense about whether a prediction proves true (Koelsch, 2014). Tensions and release may be important for a religious chorale as metaphors for sin and redemption (Koelsch, 2014).

Tension can be further modulated by a structural breach. The emotional effects of the violations of predictions, which can be treated in analogy to the free energy of a system (Friston and Friston, 2013) includes surprise. Irregular unexpected chord functions, with rating of felt tensions, evoke skin conductance responses, activity changes in the amygdala and the orbitofrontal cortex while listening to a piece of classical piano music (see Koelsch, 2014).

Anticipatory processes can also be evoked by structural cues, for example by a dominant in a Bach chorale with a high probability being followed by a tonic (see Figure 3), or a dominant seventh chord which has a high probability for being followed by a tonic, thus evoking the anticipation of release. Such anticipation of relaxation might envolve dopaminergic activity in the dorsal striatum (Koelsch, 2014).

Another effect arising from music is emotional contagion. Music can trigger psychological processes that reflect emotion: “happy” music triggers the zygomatic muscle for smiling, together with an increase in skin conductance and breathing rate, whereas “sad” music activates the corrugator muscle. Interestingly, there seems to be an acoustic similarity between expression of emotion in Western music and affective prosody (see Koelsch, 2014).

### Experimental procedures for tomographic imaging of emotion in the brain

#### Magnetic resonance imaging (MRI) and functional magnetic resonance imaging (fMRI)

**Figure 4:**
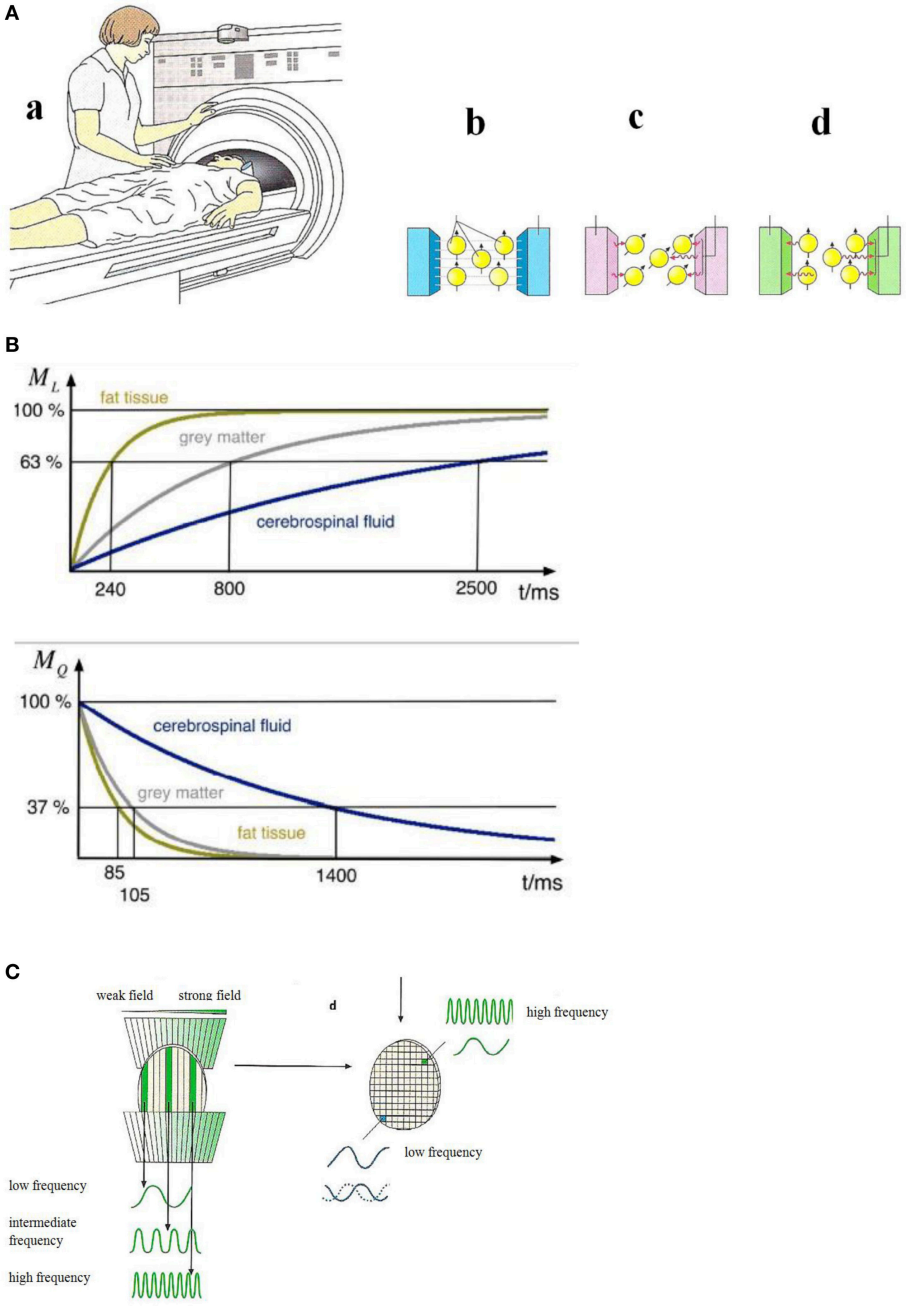
**(A)** Principles of magnetic resonance tomography (Birbaumer and Schmidt, 2010). (a) The patient is moved into the center of the MRI scanner. (b) A strong homogeneous magnetic field aligns the magnetic moments of the protons in in the patient's body. (c) An RF-pulse excites the proton magnetic moments to precession which gives rise to an alternating voltage signal in the detector. (d) After the switching-off the RF-pulse the proton magnetic moments relax to the initial orientation. The relaxation times (see **B**) are measured. Reprinted with permission from Birbaumer and Schmidt (2010) © 2010 Springer. **(B)** Nuclear magnetic relaxation times T1 (top) and T2 (bottom) of hydrogen nuclei for various biological materials (Schnier and Mehlhorn, 2013). Reprinted with permission from Schnier and Mehlhorn (2013) © 2013 Phywe Systeme. **(C)** Spatial encoding of the local magnetic resonance information (Birbaumer and Schmidt, 2010). Due to a slicing (left) and finally a three-dimensional structuring (right) by means of gradient fields, the resonance frequency and the relaxation times can be assigned to a particular pixel. Reprinted with permission from Birbaumer and Schmidt (2010) © 2010 Springer.

Magnetic resonance imaging (see Reiser et al., 2008) can show anatomy and in some cases function (fMRI). Studies on the molecular level have been reported recently (Xue et al., 2013; Liu et al., 2014). In a magnetic resonance scanner (Figure 4A) the magnetic moments of the hydrogen nuclei (protons) are aligned (Figure 4A) by a strong external magnetic field (usually 1.5 Tesla) that is generated in a superconducting coil cooled by liquid helium. Magnetic resonance of the proton magnetic moments—a quantum mechanical phenomenon—can be initiated by exciting the proton spin system to precession resonance (Figure 4A) by means of radio-frequency (RF) pulses of some milliseconds duration. This gives rise to a voltage signal with the resonance frequency ω_0_ (Larmor frequency) which decays with the relaxation times T1 (longitudinal or spin-lattice relaxation time) and T2 (transversal or spin-spin relaxation time) which are characteristic for different chemical surroundings (see Figure 4B).

A necessary condition for image generation is the exact information about the magnetic resonance signal's spatial origin. This spatial information is generated by additional site-dependent magnetic fields, called magnetic field gradients, along the three spatial axes. Due to these field gradients—much smaller in magnitude than the homogeneous main field—the magnetic field is grid-like (see Figure 4C) slightly different in each volume element (voxel). As a consequence, the application of an RF pulse with the frequency ω' excites only the nuclear magnetic moment ensemble in voxels where the Larmor frequency ω_0_—given by the local magnetic field strength—matches the resonance condition. The signal intensity which is determined by the number of nuclear spins and the relaxation times characteristic for the particular tissue (Figure 4B) is assigned in this spatial encoding procedure to an element (pixel) in the three-dimensional image. The MRI scanner (Figure 4A) comprising the homogeneous magnetic field, the RF systems, and the gradient fields is controlled by a computer including fast Fourier-transform algorithms for frequency analysis.

Functional magnetic resonance imaging (fMRI) is based on the effect that in the case of activation of neurons by, e.g., musical stimuli, an oxygen (O_2_)-enrichment occurs in oxyhemoglobin which gives rise to an enhancement of the relaxation time T2 (Birbaumer and Schmidt, 2010) of the protons of this molecule and an enhancement of the magnetic resonance signal. This effect which enables active brain areas to be imaged is called BOLD (blood oxygen level dependent) effect.

By an increase of the magnetic field strength, the signal-to-noise ratio and thereby the spatial resolution can be enhanced.

#### Positron emission tomography (PET)

**Figure 5:**
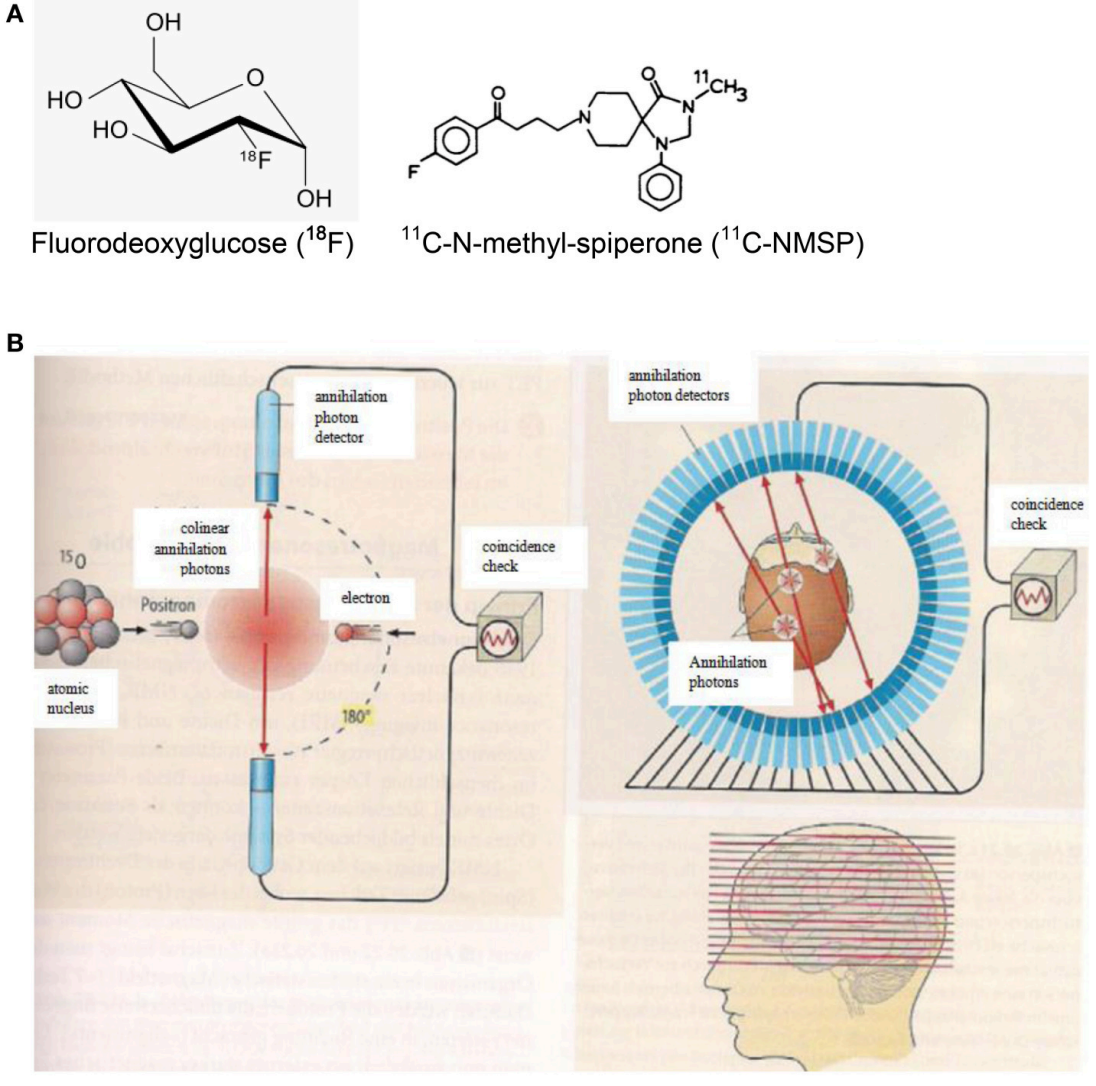
**(A)** Chemical formulae of two compounds doped with the positron emitters ^18^F (left. http://de.wikipedia.org/wiki/Fluordesoxyglucose; 19.12.14) and ^11^C (right; http://www.ncbi.nlm.nih.gov/books/NBK23614/ 19.12.14) for PET scans. **(B)** Principles of positron emission tomography (PET). Left: A positron is emitted from a radioactive nucleus and annihilated with electrons of the tissue emitting two colinear annihilation photons which are monitored by radiation detectors and checked for coincidence. Right: Multi-detector PET scanner taking images (slices) of the concentration of positron emitting isotopes in the brain and thereby measuring the emotional activity of brain sections (Birbaumer and Schmidt, 2010). Reprinted with permission from Birbaumer and Schmidt (2010) © 2010 Springer.

PET imaging is based on the annihilation of positrons with electrons of the body. The positrons are emitted from proton-rich radioactive atomic nuclei (see Table 1) which are embedded in specific biomolecules (Figure 5A). The positron-electron annihilation process gives rise to two high-energy (0.511 MeV) annihilation photons (Figure 5B) which can be monitored by radiation detectors around the body of the patient and thereby identify the site of the radioactive element. In a PET camera or PET scanner many detectors are implemented (Figure 5B) allowing for tomographic imaging with good spatial resolution of about 4 mm.

**Table 1:** PET isotopes produced by high energy protons in a cyclotron accelerator.

	**Half live (min)**	**Production reaction**	**Frequent application**	**Max. e^+^- energy (MeV)**	**Average e^+^- range (mm H_2_O)**
^11^C	20.4	^11^B(10MeVp,n)^11^C	Postsynaptic receptors	0.96	1.1
^15^O	2.0	^14^N(10MeVd,n)^15^O	Oxygen consumption	1.73	2.8
^18^F	109.7	^18^O(10MeVp,n)^18^F	Glucose metabolism	0.64	0.6

*see http://en.wikipedia.org/wiki/Positron_emission_tomography; downloaded 22.12. 14*.

Making use of fluorodeoxyglucose (^18^F-FDG) doped with the radioactive fluorine isotope ^18^F (Figure 5A), the local sugar metabolism in neurologically activated areas of the brain can be monitored (Figure 5B). After injection of ^18^F-FDG into a patient, a PET scanner (Figure 5B) can form a three-dimensional image of the ^18^F-FDG concentration in the body. For specifically probing molecular changes in postsynaptic monoamine receptors such as the dopamine receptor D_2_ and the serotonin receptor 5-HT_2A_, ^11^C-N-methyl-spiperone (11C-MNSP, Figure 5A) doped with the positron-emitting carbon isotope ^11^C can be used. It should be pointed out here that the combination of MRI/PET (Bailey et al., 2014) represents an innovative imaging modality.

### Experimental results of functional (tomographic) brain imaging (fMRI, PET)

#### Movements during listening to music

Music is a universal feature of human societies, partly owing to its power to evoke strong emotions and influence moods. Understanding of neural correlates of music-evoked emotions has been invaluable for the understanding of human emotions (Koelsch, 2014).

**Figure 6:**
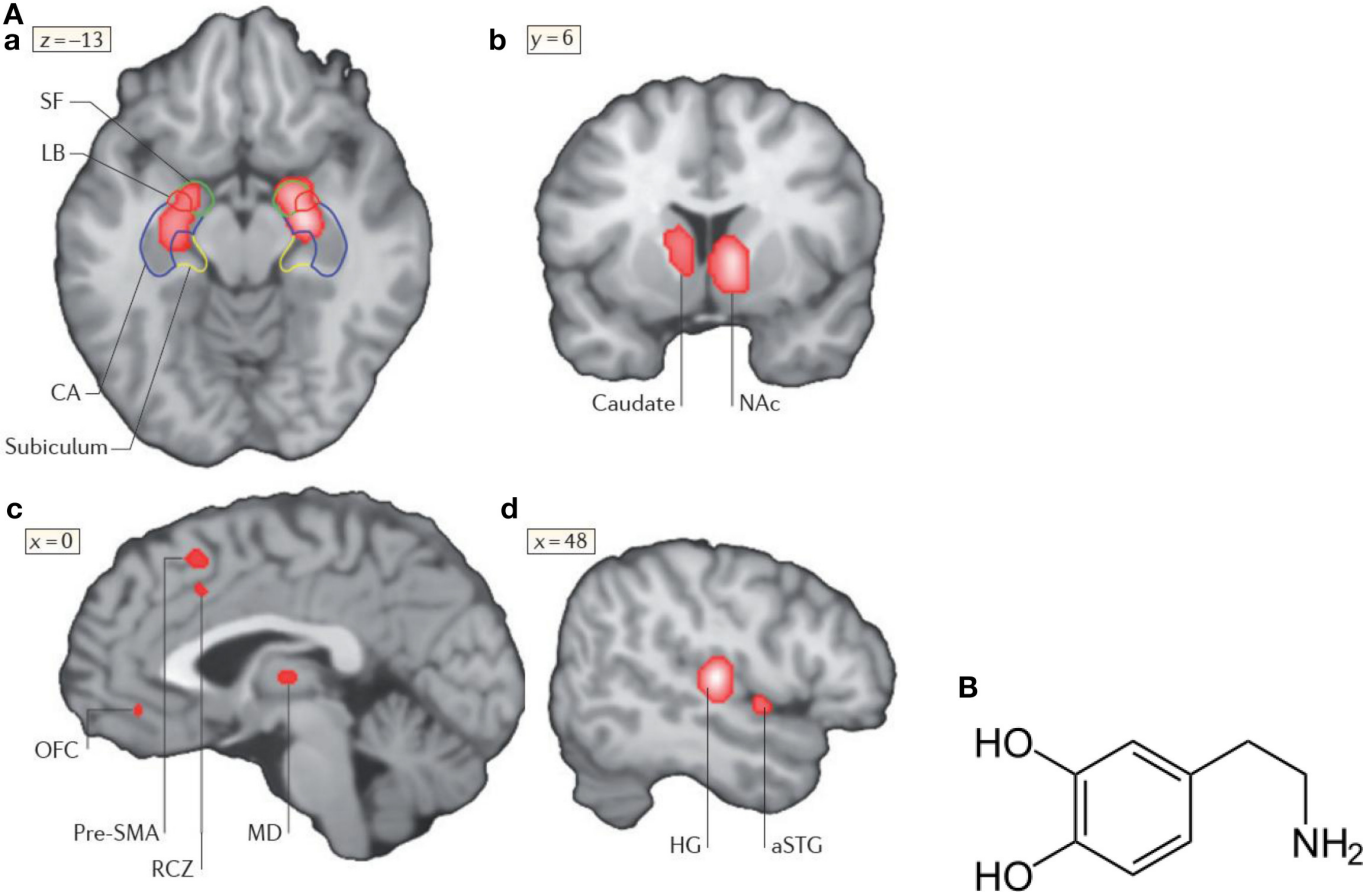
**(A)** Neural correlates of music-evoked emotions. A meta-analysis of brain-imaging studies that shows neural correlates of music-evoked emotions. A meta-analysis is a statistical analysis of a lager set of the analyses of earlier data. The meta -analysis indicates clusters of activities derived from numerous studies (for references see Koelsch, 2014) in the amygdala (SF, LB), the hippocampal formation (a), the left caudate nucleus with a maximum in the nucleus accumbens (NAc, b), pre-supplementary motor area (SMA), rostral cingulated zone (RCZ), orbifrontal cortex (OFC), and mediodorsal thalamus (MD, c), as well as in auditory regions (Heschls gyrus HG) and anterior superior temporal gyrus (aSTG, d). Additional limbic and paralimbic brain areas may contribute to music-evoked emotions. For details see Koelsch (2014). Reprinted with permission from Koelsch (2014) © 2014 Nature Publishing Group. **(B)** Structural formula of dopamine (http://de.wikipedia.org/wiki/Dopamin) downloaded19.12.14.

Functional neuroimaging studies on music and emotion, such as fMRI and PET (see Figure 6A) show that music can modulate the activity in brain structures that are known to be crucially involved in emotion, such as the amygdala and nucleus accumbens (NAc). The nucleus accumbens plays an important role in the mesolimbic system generating pleasure, laughter, reward but also fear, aggression, impulsivity, and addiction. The mesolimbic system is additionally intensely involved in emotional learning processes. Drugs can in this system effectuate the release of the neurotransmitter dopamine (Figure 6B). Neurotransmitters such as dopamine, serotonin, adrenaline, noradrenaline, or acetylcholine are biochemicals (see Figure 6B) which diffuse across a chemical synapse, bind to a postsynaptic receptor opening a sodium ion (Na^+^) channel to transfer the excitation of a neuron to the neighboring neuron.

A meta-analysis of functional neuroimaging studies (fMRI, PET) of music-evoked emotions is shown in Figure 6A, including studies of music of intense pleasure, consonant or dissonant music, happy or sad music, joy- or fear-evoking music, muzak, expectancy violations, and music-evoked tension (for references see Koelsch, 2014).

In response to music, changes of the activity of the amygdala, the hippocampus, the right central striatum, the auditory cortex, the pre-supplementary motor area, the cingulate cortex, and the orbitofrontal cortex are observed (Figure 6A). In the following, the role of the amygdala, the nucleus accumbens and the hippocampus in music-evoked emotion is briefly discussed in more detail.

##### Amygdala

The amygdala is central in the emotion network and can regulate and modulate this network. It processes emotions such as happiness, anxiety, anger, annoyance, and, additionally assesses the impression of facial expression and thereby contributes to communication, social behavior, and memory (Kraus and Canlon, 2012). It, moreover, releases a number of neurotransmitters such as dopamine and serotonin, and effectuates reflexes such as being scared (Kraus and Canlon, 2012). The amygdala receives input from the central auditory system (Kraus and Canlon, 2012) and the sensory systems, and its pathways to the hypothalamus affect the sympathetic neuronal system for the release of hormones via the hypothalamus-pituitary-adrenal (HPA)-axis but also the parasympathetic neuronal system (Kraus and Canlon, 2012). The hormone cortisol and the neuropeptide endorphine have been observed in musical tasks 20 years ago (see Kreutz et al., 2012).

**Figure 7:**
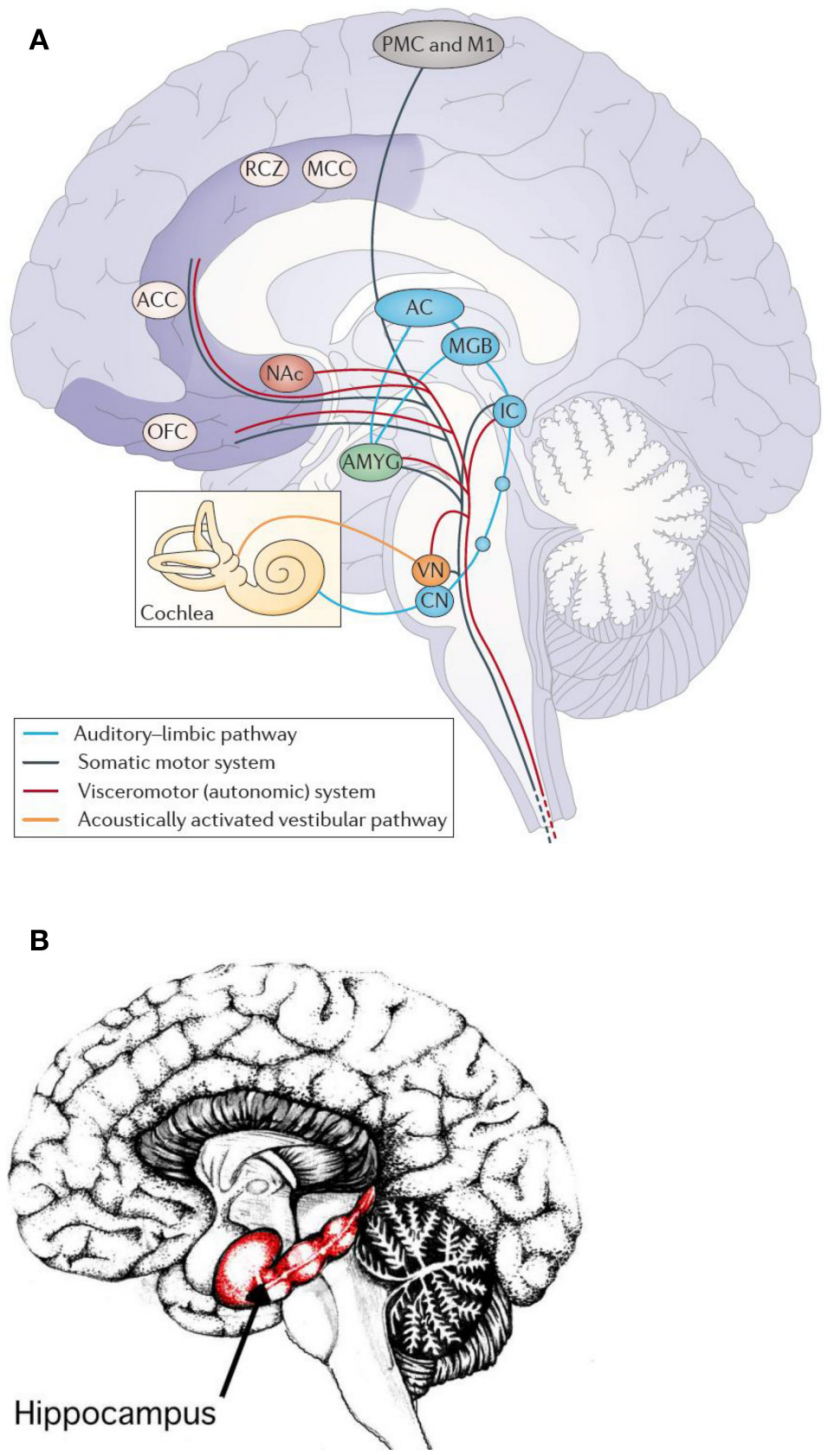
**(A)** Main pathways underlying autonomic and muscular responses to music. The cortex (AC) also projects to the orbifrontal cortex (OFC) and the cingulated cortex (projections not shown). Moreover, the amygdala (AMYG), the OFC and the cingulated cortex send numerous projections to the hypothalamus (not shown) and thus also exert influence on the endocrine system. ACC, anterior cingulate cortex; CN, cochlear nuclei; IC, inferior colliculus; M1, primary motor cortex; MCC, middle cingulate cortex; MGB, medial geniculate body; NAc, nucleus accumbens; PMC, premotor cortex; RCZ, rostral cingulated zone; VN, vestibular nuclei (Koelsch, 2014). Reprinted with permission from Koelsch (2014) © 2014 Nature Publishing Group. **(B)** Hippocampus. Reprinted with permission from Annie Krusznis © 2016.

Fear conditioning is mediated by synaptic plasticity in the amygdala (Koelsch et al., 2006). It may affect the auditory cortex and its plasticity (learning) by a thalamus-amygdala-cullicular feedback circuit (Figure 7A). Neuronal pathways between the hippocampus and the amygdala allow for a direct interaction of emotion and declarative verbally describable memory and vice versa (Koelsch et al., 2006).

**Figure 8:**
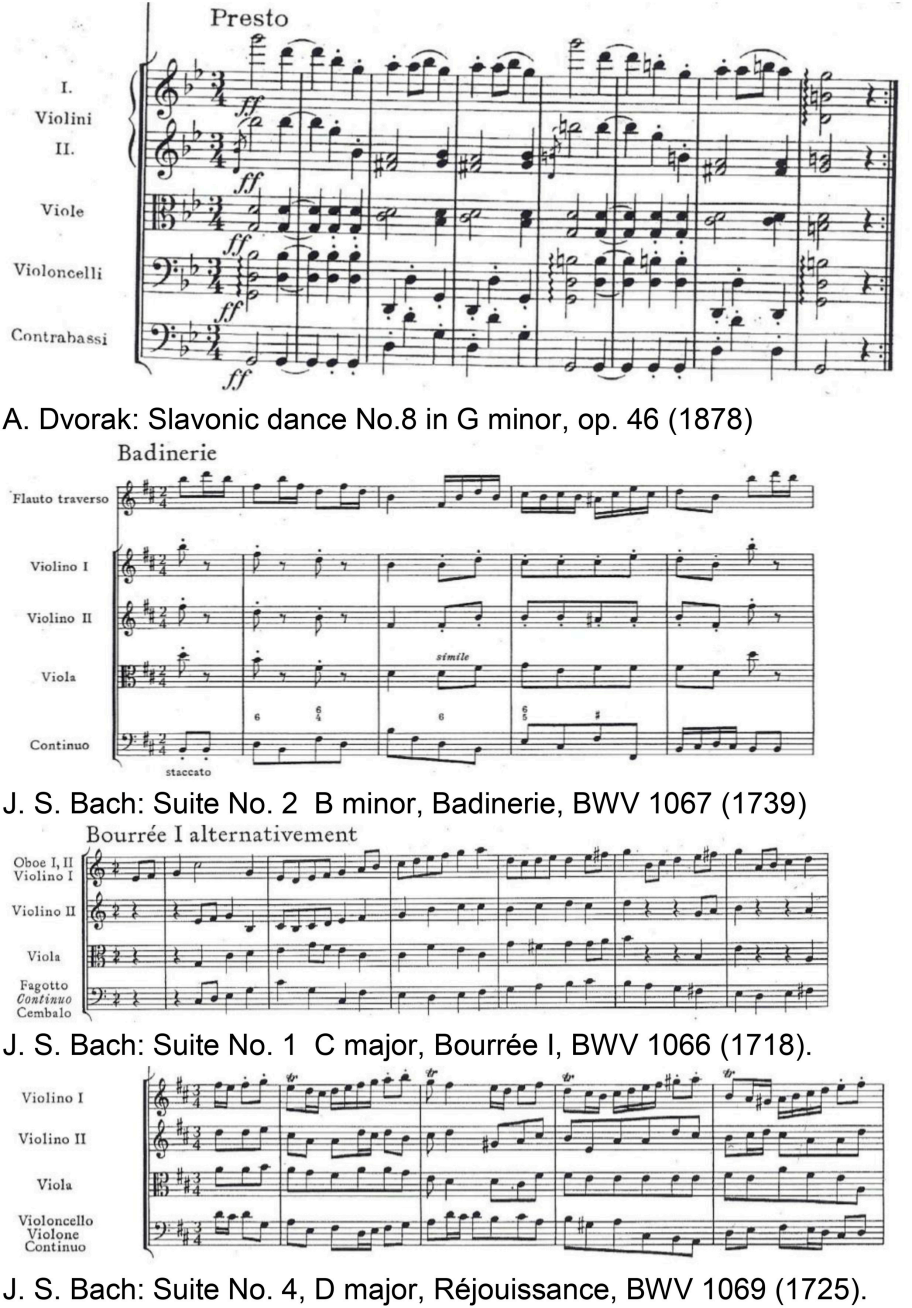
Joyful instrumental dance-tunes of major-minor tonal music by Dvorak (1955) and Bach (1967) used from commercially available CDs as pleasant stimuli in Koelsch et al. (2006). Reprinted with permission from Bach (1967) © 1967 Bärenreiter.

The superficial amygdala is sensitive to faces, sounds, and music that is perceived as pleasant or joyful. Functional connections between the superficial amygdala, the nucleus accumbens (Figure 7A), and the mediodorsal thalamus are stronger during joy-evoking music than during fear-evoking music. The laterobasal amygdala shows activity changes during joyful or sad music. The connection of the amygdala to the hypothalamus affects the sympathetic neuronal system for the release of corticosteroid hormones via the HPS-axis and also affects the parasympathetic neural system (Kraus and Canlon, 2012). Functional magnetic resonance imaging (fMRI) (Koelsch et al., 2006) evidenced music-induced activity changes in the amygdala, ventral striatum and the hippocampal formation without the experience of “chills.” The study compared the brain responses of joyful dance-tunes by A. Dvorak and J. S. Bach (Figure 8) played by professional musicians with responses to electronically manipulated dissonant (unpleasant) variations of these tunes. Unpleasant music induced increases of the blood-oxygen-level dependent (BOLD) signals in the amygdala and the hippocampus in contrast to pleasant music giving rise to BOLD decreases in these structures. In a PET experiment (Blood and Zatorre, 2001) the participants' favorite CD music was used in order to induce “chills” or “shivers down the spine.” Increased chill intensity was observed in brain regions ascribed to reward and emotion such as the nucleus accumbens (NAc), in the anterior cingulate cortex (ACC) and the orbitofrontal cortex (see Figure 7A). Decreases of the blood flow were observed in the amygdala and the anterior hippocampal formation with increasing chill intensity.

These observations demonstrated the modulation of the activities of the brain core structures ascribed to emotion processing by music. Furthermore, they gave direct support to the phenomenological efforts in music-therapeutic approaches for the treatment of disorders such as depression and anxiety because these disorders are partly ascribed to dysfunctions of the amygdala and presumably of the hippocampus (Koelsch and Stegemann, 2012) (see section Musical Therapy for Psychiatric or Neurologic Impairments and Deficiencies in Music Perception).

##### Nucleus accumbens (NAc)

The activities observed by functional neuroimaging in this brain section (see Figure 7A) are initiated by “musical frissons,” involving experiences of shivers or goose bumps. This brain section is sensitive to primary rewards (food, drinks, or sex), consuming the rewards, and to addiction. This shows that music-evoked pleasure is associated with the activation of a phylogenetically old reward network that functions to ensure the survival of the individual and the species. The network seems to be functionally connected with the auditory cortex: while listening to music the functional connectivity between the nucleus accumbens and the auditory cortex predicts whether individuals will decide to buy a song (Salimpoor et al., 2013).

A PET study on musical frissons (Blood and Zatorre, 2001) making use of the radioactive marker ^11^C-raclopride to measure the release of the neurotransmitter dopamine at synapses indicated that neural activity in the ventral and dorsal striatum involves increased dopamine availability, probably released by dopaminergic neurons in the ventral tegmental area (VTA). This indicates that music-evoked pleasure is associated with activation of the mesolimbic dopaminergic reward pathway.

##### Hippocampus

A number of studies on music-evoked emotions has reported activity changes in the hippocampus (see Figure 7B), in striking contrast to the monetary or erotic rewards which do not activate the hippocampus (see Koelsch, 2014). This suggests that music-evoked emotions are not related to reward alone. Hippocampal activity was associated in some studies with music-evoked tenderness, peacefulness, joy, frissons or sadness and both, positive or negative emotions (for references see Koelsch, 2014). There is mounting evidence that the hippocampus is involved in emotion due to its role in the hippothalamus-pituitary-adrenal (HPA) axis stress response. The hippocampus appears to be involved in music-evoked positive emotions that have endocrine effects (see section Psychoneuroendocrinology—Neuroendocrine and Immunological Markers) associated with a reduction of emotional stress effectuated by a lowering of the cortisol (C_21_H_30_O_5_) level which controls the carbon hydrate, fat, and protein metabolisms.

Another emotional function of the hippocampus in humans, beyond stress regulation, is the formation and maintenance of social attachments, such as, e.g., love. The evocation of attachment-related neurological activities by music appears to confirm the phenomenologically observed social functions of music establishing, maintaining, and strengthening social attachments. In this sense, music is directly related to the fulfillment of basic human needs, such as contact and communication, social cohesion and attachment (Koelsch, 2014). Some researchers even speculate that the strengthening of inter-individual attachments could have been an important adaptive function of music in the evolution of humans (Koelsch, 2014).

The prominent task of the hippocampal-auditory system is the long-term auditive memory. The downloading from the music memory activates the hippocampus predominantly on the right hemisphere (Watanabe et al., 2008). The hippocampus is, due to its projections to the amygdala, also involved in the emotional processing of music (Mitterschiffthaler et al., 2007). fMRI studies show an activation of the right hippocampus and the amygdala by sad music but not by happy or neutral music (Koelsch et al., 2006). Functional neuroimaging studies investigated how music influences and interacts with the processing of visual information (see Koelsch, 2014). These studies show that a combination of films or images with music expressing joy, fear, or surprise increase BOLD responses in the amygdala or the hippocampus (see Koelsch, 2014).

The hippocampus finds projections from the frontal, temporal and parietal lobes, as well as from the parahippocampal and the perirhinal cortices. The amygdala can modify the information storage processes of the hippocampus but, inversely, the reactions generated in the amygdala by external stimuli can be influenced by the hippocampus. These synergetic effects can contribute to the long-term storage of emotional events which is supported by the plasticity of the two units, enabling the acquisition of experience.

The degree of overlap between music-evoked emotions and so-called everyday emotions remains to be specified. Some musical emotions may appear in everyday life, such as surprise or joy. Some emotions are sought in music because they might be rare in everyday life, such as transcendence or wonder and some so-called moral emotions of everyday life, such as shame or guilt are lacking in music (Koelsch, 2014).

**Figure 9:**
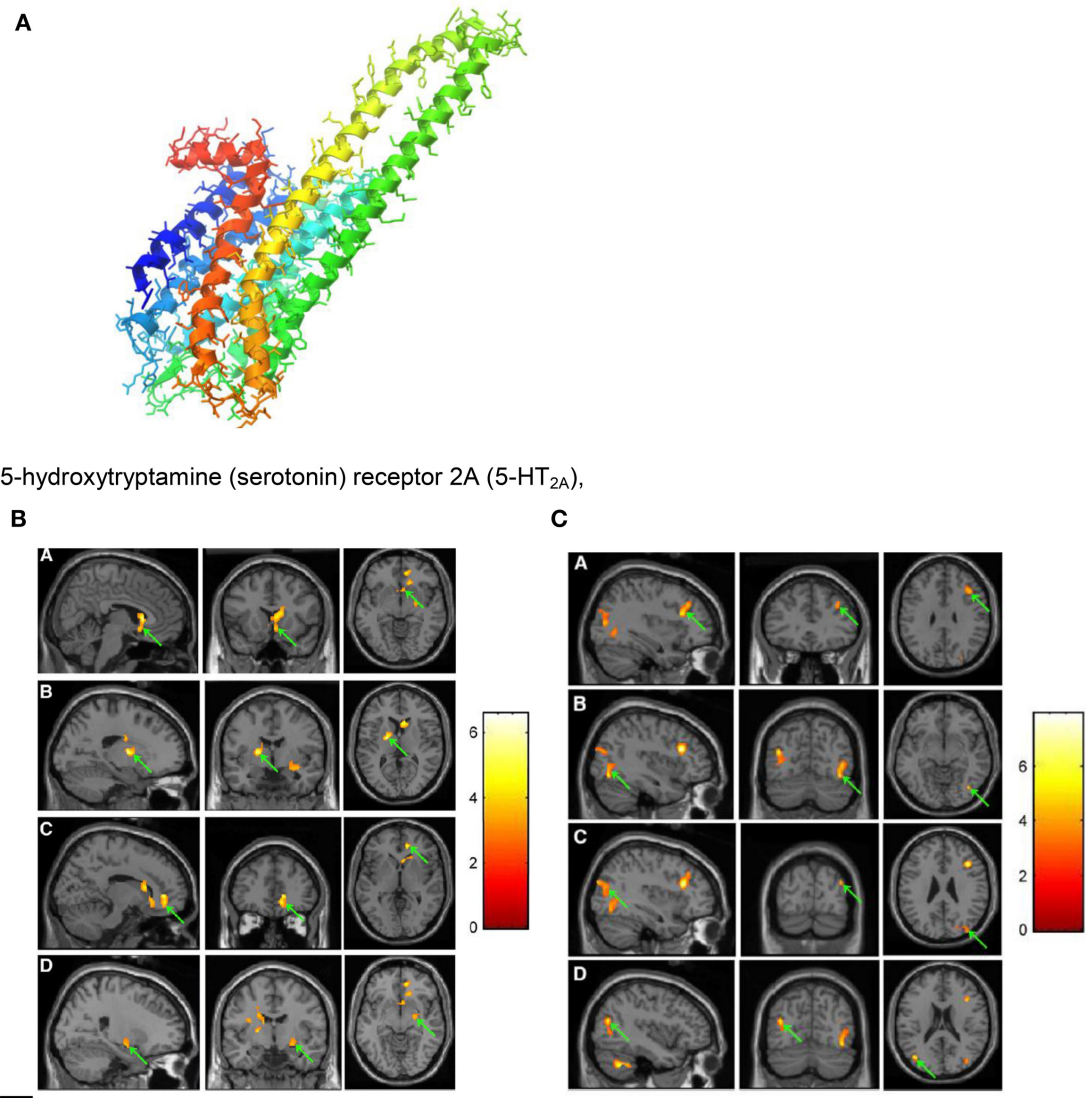
**(A)** 5-hydroxytryptamine (serotonin) receptor 2A (5-HT_2A_), G protein coupled; diameter of the protein alpha-helix ~0.5 nm https://en.wikipedia.org/wiki/5-HT2A_receptor downloaded 4. 10. 2016. **(B)** PET images showing decrease in ^11^C-NMSP binding clusters (arrows) in a subject listening to frightening music: right caudate head, right frontal subgirus, and right anterior cingulated (A); left lateral globus pallidus and left caudate body (B); right anterior cingulated (C); and right superior temporal gyrus, right claustrum, and right amygdala. (D) (Zhang et al., 2012). Reprinted with permission from Zhang et al. (2012) © 2012 SNMMI. **(C)** PET images showing increase in 11C-NMSP binding clusters (arrows) in a subject listening to frightening music: right frontal lobe and middle frontal gyrus (A); right fusiform gyrus and right middle occipital gyrus (B); right superior occipital gyrus, right middle occipital gyrus (C); and left middle temporal gyrus (D) (Zhang et al., 2012). Reprinted with permission from Zhang et al. (2012) © 2012 SNMMI.

The molecular level of music-evoked neural processes can be achieved by making use of PET scans employing biomolecules doped with radioactive positron emitters. By using ^11^C-N-methyl-spiperone (^11^C-NMSP, see Figure 5A) as an antagonist binding the postsynaptic dopamine receptor 2 (D_2_) and the serotonin receptor 5-hydroxytriptamine2A (5-HT_2A_, see Figure 9A), acute changes of these neurotransmitter receptors in response to frightening music could be demonstrated (Zhang et al., 2012). Thus, the binding of ^11^C-NMSP directly reflects the postsynaptic receptor level. Because the antagonist ^11^C-NMSP binds predominantly D_2_ in the striatum and 5-HT_2A_ in the cortex the antagonist can be used to map these receptors directly and simultaneously in the same individual (Watanabe, 2012). It is hypothesized (Zhang et al., 2012) that emotional processing of fear is mediated by the D_2_ and the 5-HT_2A_ receptors. Frightening music is reported (Zhang et al., 2012) to rapidly arouse emotions in listeners that mimic those from actual life-threatening experiences.

However, studies of the underlying mechanisms for perceiving danger created by music are limited. The musical stimulus in the investigations on frightening music (Zhang et al., 2012) discussed here was selected from the Japanese horror film *Ju-On* which is widely accepted as one of the scariest and most influential movies ever made (Shimizu, 2004). The film music (see *The Grudge theme song* https://www.youtube.com/watch?v=1dqjXyIu02s) has been composed by Shiro Sato.

For the PET scans (see Figures 9B,C) ^11^C-NMSP-activities of 740 MBq (20 mCi) were used. In the course of frightening music significant decreases in ^11^C-NMSP binding was observed in the limbic and paralimbic brain regions in four clusters (Figure 9B): In the right caudate head, the right frontal subgyral region, and the right anterior cingulate region (A); the left lateral globus pallidus and left caudate body (B); the right anterior cingulate region (C); and the right superior temporal gyrus, right claustrum, and right amygdala (D). Increased ^11^C-NMSP accumulation (Figure 9C) was found in the cerebral cortex, in the right frontal lobe and the middle frontal gyrus (A); the right fusiform gyrus and the right middle occipital gyrus (B); the right superior occipital gyrus, the right middle occipital gyrus, and the superior occipital gyrus (C); and the left middle temporal gyrus (D).

The decrease in the caudate nucleus in response to frightening music indicates that frightening music triggers a downregulation of postsynaptic D_2._ This suggests that the caudate nucleus is involved in a wide range of emotional processes evoked by music (Zhang et al., 2012). The finding that the ^11^C-NMSP binding decreases significantly (Figure 9B) during frightening music demonstrates the musical triggering of the monoamine receptors in the amygdala. It is assumed (Zhang et al., 2012) that changes of ^11^C-NMSP binding (Figures 9B,C) mainly reflect 5-HT_2A_ levels in the cortex, where 5-HT_2A_ overdensity is thought to be involved in the pathogenesis of depression (Eison and Mullins, 1996).

It should be additionally pointed out that the ^11^C-NMSP PET study (Zhang et al., 2012) found the right hemisphere to have superiority in the processing of auditory stimuli and the defense reaction.

#### Movements of performing musicians

**Figure 10:**
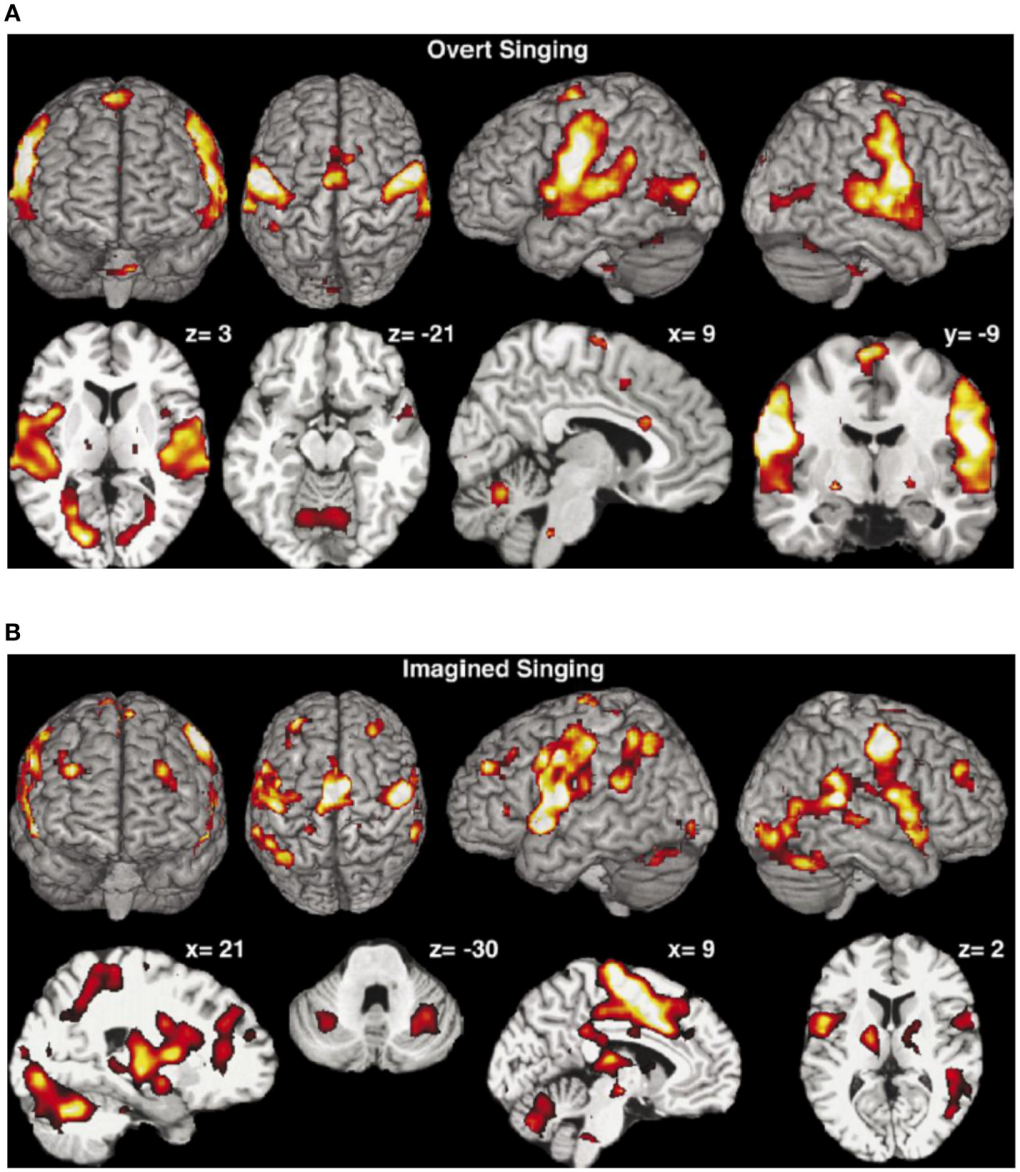
**(A)** Overt singing. The activation maps show activations of the bilateral sensorimotor cortex and the cerebellum, the bilateral auditory cortex, Broca's and Wernicke's areas, medulla, thalamus, and ventral striatum but also ACC and insula were activated. Coordinates of cuts are given above each slice (Kleber et al., 2007). Reprinted with permission from Kleber et al. (2007) © 2007 Elsevier. **(B)** Mental rehearsal of singing (imaginary singing). Activation of typical imagery regions such as sensorimotor areas (SMA), premotor cortex areas, thalamus, basal ganglia, and cerebellum. Areas processing emotions showed intense activation (ACC and insula, hippocampus, amygdala, and ventrolateral prefrontal cortex). Coordinates of cuts are given above each slice (Kleber et al., 2007). Reprinted with permission from Kleber et al. (2007) © 2007 Elsevier.

Brain activation of professional classical singers has been monitored by fMRI during overt singing and imagined singing of an Italian aria (Kleber et al., 2007). Overt singing (Figure 10A) involved bilateral primary (A1) and secondary sensorimotor areas (SMA) and auditory cortices with Broca's and Wernike's areas but also areas associated with speech and language.

Activation in the gyri of Heschl occurred in both hemispheres, together with the subcortical motor areas (cerebellum, thalamus, medulla and basal ganglia) and slight activation in areas of emotional processing (anterior cingulate cortex, anterior insula). Imagined singing (Figure 10B) effectuated cerebral activation centered in fronto-parietal areas and bilateral primary and secondary sensorimotor areas. No activation was found in the primary auditory cortex or in the auditory belt area. Regions processing emotion showed intense activation (anterior cingulate cortex—ACC, insula, hippocampus, and amygdala).

Performing music in one's mind is a technique commonly used by professional musicians to rehearse. Composers write music regardless of the presence of a musical instrument, as, e.g., Mozart or Schubert did (see Kleber et al., 2007). Singing of classical music involves technical-motor and emotional engagement in order to communicate artistic, emotional, and semantic aspects of the song. A tight regulation of pitch, meter, and rhythm as well as an increased sound intensity and vocal range, vibrato and a dramatic expression of emotion are indispensible. Motor aspects of these requirements are reflected in a fine laryngeal motor control and a high involvement of the thoracic muscles during singing. The aria used in this study (Kleber et al., 2007) comprises text, rhythm, and melody which make the bilateral activation of A1 plausible.

For the study of music-evoked emotions during performing in the fMRI scanner the bel canto aria *Caro mio ben* by Tommaso Giordani (1730-1806) has been used (Kleber et al., 2007).

Interestingly, most areas involved in motor processing were activated both during overt singing and imaginary singing, a finding that may demonstrate the significance of imagined rehearsal. The basal ganglia which were active in both overt and imaginary singing may be involved in the modulation of the voice. The overt singing task activated only the ACC and the insula which were both also activated during imaginary singing. The ACC is involved in the recall of emotions (Kleber et al., 2007)—a capability which is important for both overt and imaginary performance. The activation of the insula seems to reflect the intensity of the emotion. The amygdala which was only activated by imagined singing is known to be involved in passive avoidance or approach tasks. This is reported (Kleber et al., 2007) to be consistent with the observation that the amygdala was not active during overt singing. Imagined singing activated a large fronto-parietal network, indicating increased involvement of working memory processes during mental imagery which in turn may indicate that imagined singing is less automatized than overt singing (Kleber et al., 2007). Areas processing emotions showed also enhanced activation during imagined singing which may reflect increased emotional recall during this task.

An overview of the sensory-motor control of the singing voice has been given based on fMRI research of somatosensory and auditory feedback processing during singing in comparison to theoretical models (Zarate, 2013).

Movement organization that enables skilled piano performance has been recently reviewed, including the advances in diagnosis and therapy of movement disorders (Furuya and Altenmüller, 2013).

### Psychoneuroendocrinology—neuroendocrine and immunological markers

Psychoneuroendocrinology (PNE) aims at the study of the musical experiences leading to hormonal changes in the brain and the body. These effects may be similar to those effectuated by pharmacological substances. In addition to investigating psychiatric illnesses and syndromes, PNE investigates more positive experiences such as the neurobiology of love (see Kreutz et al., 2012). In contrast to the neuronal system which transmits its messages by electrical signals, the endocrinal system makes use of biomolecules, such as hormones in order to communicate with the target organs which are equipped with specific receptors for these hormones (see Birbaumer and Schmidt, 2010).

For considering the neuroendocrine and immunological molecular markers which could be released during music-evoked emotion, the three interrelated systems regulating hormonal stress responses should be briefly introduced:

The hypothalamic-pituitary-adrenocortical axis (HPA). This axis is initiated by a stimulus in the brain area of the hypothalamus giving rise to the release of the corticotropin releasing factor (CRF) which in turn leads to the release of adrenocorticotropic hormone (ACTH) and beta-endorphin from the pituitary into the circulation. ACTH then stimulates the synthesis and release of cortisol and of testosterone from the adrenal cortex.

**Figure 11:**
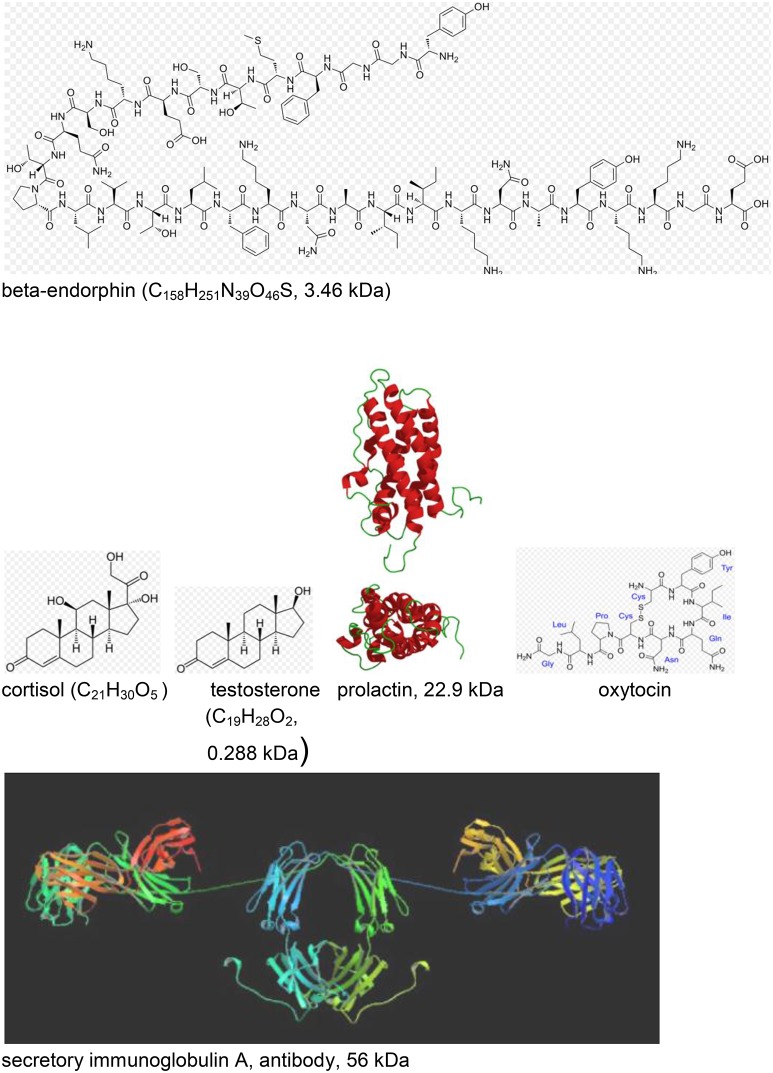
Neuroendocrine and immunological molecular markers released during music- evoked emotion (see Kreutz et al., 2012). The molecular masses are given in kDa = 1.66 × 10^−24^ kg. http://en.wikipedia.org/wiki/Beta-endorphin#mediaviewer/File:Betaendorphin.png; http://de.wikipedia.org/wiki/Cortisol; http://de.wikipedia.org/wiki/Testosteron; http://de.wikipedia.org/wiki/Prolaktin; http://de.wikipedia.org/wiki/Oxytocin; http://en.wikipedia.org/wiki/Immunoglobulin_A downloads 20.12.2014.

Beta-endorphin (see Figure 11) is a hormone where increased concentration levels are associated with situative stress. Delivering special relaxation music to coronary patients leads to significant decrease of beta-endorphin concentration with a simultaneous reduction of blood pressure, anxiety and worry. Music therapy can also be effective before and during surgeries in operating theaters, again due to a reduction of the beta-endorphin level (see Kreutz et al., 2012).

Cortisol (see Figure 11) is a hormone where high levels of concentration are associated with psychological and physiological stresses. Listening to classical choral, meditative, or folk music significantly reduces the cortisol level, however, increases have been detected for listeners exposed to Techno (see Kreutz et al., 2012). Individual differences were evidenced in listening experiments where music students responded with increases and biology students with decreases of the cortisol levels. Changes of the cortisol concentration can also be induced by actively singing. In clinical context, exposure to music has been shown to reduce cortisol levels during medical treatment. In gender studies cortisol reductions were found in females in contrast to males, exhibiting increases. Little is known about the sustainability of these effects over a longer period of time (see Kreutz et al., 2012).

Testosterone (see Figure 11), a sex hormone, appears to be of particular relevance to music. Darwin (1871; see Kreutz et al., 2012) suggests music as originating from sexual selection. Female composers showed above average and male composers below average testosterone levels which has initiated discussions whether physiologically androgynous individuals are on a higher level of creativity.

Secretory immunoglobulin A (sIgA; see Figure 11) is an antibody considered as a molecular marker of the local immune system in the respiratory tract and as a first line of defense against bacterial and viral infections. High levels of sIgA may exert positive effects and low levels may be characteristic for chronic stress. Significant increases of sIgA concentrations were observed in response to listening to relaxation music or musak. Increases of the sIgA concentration were observed from rehearsal to public performance of choral singers (Kreutz et al., 2012).

Another study investigated the concentration of prolactin (see Figure 11) while listening to music of Hans-Werner Henze. The concentration of prolactin which is a hormone with important regulatory functions during pregnancy decreased in response to Henze (Kreutz et al., 2012).

It should be summarized that the neuroendocrine changes reflecting the psychophysiological processes in response to music appear to be complex but might promise favorable effects with respect to health implications deserving enhanced research activities.

The simpatho-adrenomedullary system is part of the sympathetic nervous system executing fight and flight responses. By, e.g., stress activation, norepinephrine is released. Sympathetic enervations of the medulla of the adrenal glands give rise to the secretion of the catecholamines (dopamine, epinephrine, norepinephrine). Since this works by nervous operation of the adreanal gland it responds much faster than the HPA which is regulated by hormonal processes.

The endogeneous opioid system is related to the HPA axis and can influence the ACTH and cortisol levels in the blood (see Kreutz et al., 2012). None of these three responses is specific to one kind of challenge and the response delays vary to a great deal.

There is an increasing interest in PNE research for studying musical behavior due to the increasing specificity of neuroendocrinological research technologies. It is likely that musical behaviors significantly influence neurotransmitter processes.

Whether music processing can be associated with the processing of, e.g., linguistic sound is a matter of debate (Kreutz et al., 2012). However, functional imaging brain studies suggest that the perception of singing is different of the perception of speech since singing evokes stronger activations in the subcortical regions which are associated with emotional processing (see Kreutz et al., 2012).

Experiments are suggested (Chanda and Levitin, 2013) that aim to uncover the connection between music, the neurochemical changes in the following health domains

- Reward, motivation, and pleasure,
- Stress and arousal,
- Immunity, and
- Social affiliation,

and the neurochemical systems

- Dopamine and opioids,
- Cortisol, adrenocorticotropic hormone (ACTH)
- Serotonin, and
- And the “love” drug oxytocin (see Figure 11).

### Electro- and magnetoencephalography (EEG, MEG)

#### Electroencephalography (EEG) and event-related brain potentials (ERP)

This technique yields valuable information on the brain—behavior relationship on much shorter time scales (ms) than tomography, however, with limited spatial information.

**Figure 12:**
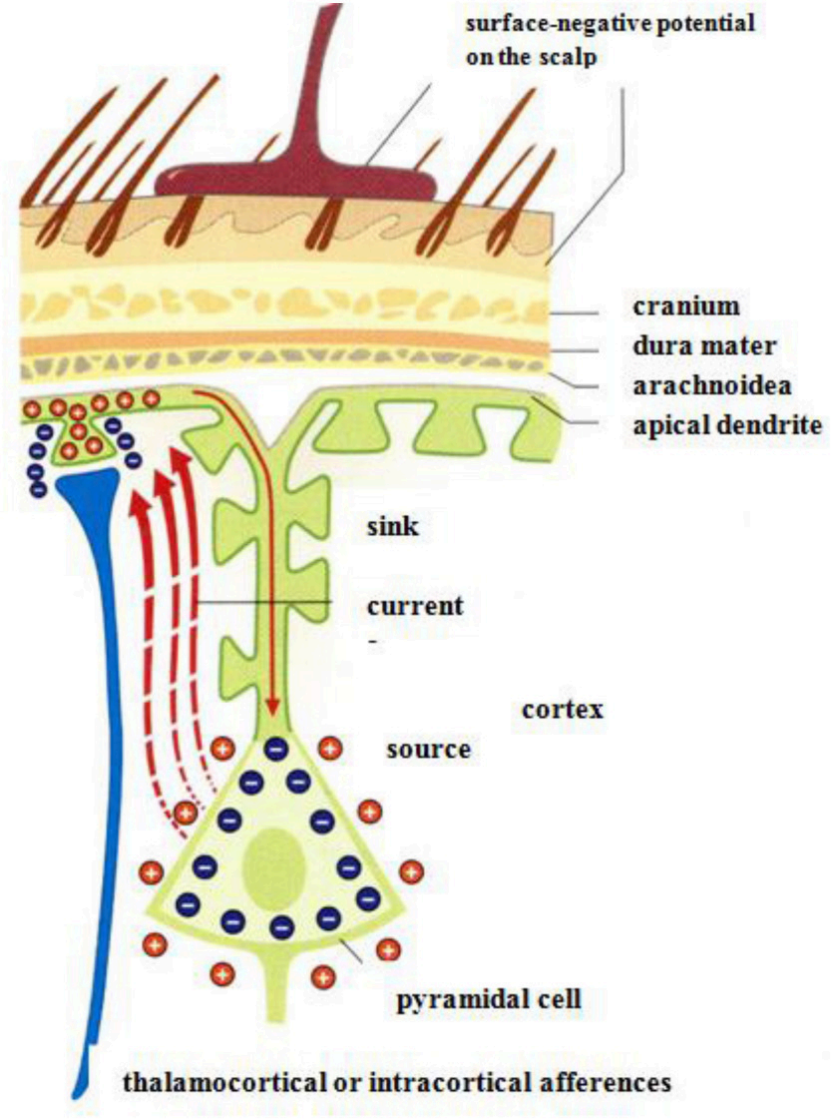
Negative surface slow brain potentials on the skalp are generated by extracellular currents (red dashed arrows) which arise due to the electrical activation of apical dendrites by thalamocortical afferences (Birbaumer and Schmidt, 2010). Reprinted with permission from Birbaumer and Schmidt (2010) © 2010 Springer.

Measurements of electrical potentials are performed making use of an array of voltage probes on the scalp. The EEG arises due to electrical potential oscillations in the brain, i.e., by excitatory postsynaptic potentials. Cortical afferences of the thalamus activate the apical dendrities (see Figure 12). Compensating extracellular electrical currents (Figure 12) generate measurable potentials on the scalp with characteristic oscillations in the frequency range of about 4–15 Hz (Birbaumer and Schmidt, 2010). Event-related brain potentials (ERPs) are of particular interest in the present context of considering music-evoked emotions (Neuhaus, 2013). By synchronized averaging of many measurements, the ERPs are extracted from noise showing a sequence of characteristic components which can be ascribed to separate phases of cognitive processes. Slow negative potentials (100–600 ms) are thought to be generated by cortical cholinergic synapses with high synchronization of pulses at the apical dendrites (see Figure 12). Positive potentials may be due to a decrease of the synchronization of the thalamic activity (Birbaumer and Schmidt, 2010).

The interpretation of single ERP components as correlates of processing specific information is on a phenomenological stage. Up to 300 ms the components are ascribed to unconscious (autonomous) processing. Changes of consciousness can be attributed to components from 300 ms and higher (Birbaumer and Schmidt, 2010).

An impressive neurocognitive approach to musical form perception has been presented recently by ERP studies (Neuhaus, 2013). The study investigates the listeners' chunking abilities of two eight-measure theme types AABB and ABAB for pattern similarity (AA) and pattern contrast (AB). In the experiments a theme type of eight measures in length (2+2+2+2), often found in the Classical and Romantic periods, was used. In addition to behavioral rating considerations, ERP measurements were performed while non-musicians listened. The advantage of ERP, compared to the more direct neuroimaging techniques such as PET and fMRI, is the good time resolution in range of about 10 ms.

The experiments were performed on 20 students without musical training. The tunes were presented in various transpositions so that the tonality has not to be considered as an independent parameter. Each melody of the AABB or ABAB form types used the harmonic scheme tonic—dominant—tonic. The melodies with an average duration of 10.8 s and form part length of 2.7 s were presented from a programmable keyboard with a tempo of 102.4 BPM. The brain activity was measured making use of 59 Ag/AgCl electrodes with an impedance below 5 Ω.

In the behavioral studies the sequence ABAB is more often assessed as non-sequential than the sequence AABB. The tendency to recognize chunk form parts was high with the two following aspects coinciding: Rhythmic contrasts in A and B and when the melodic contour was upward- downward.

In grand average ERPs, an anterior negative shift N300 for immediate AA sequences as well as for non-immediate repetitions ABA or ABAB of similar form parts was observed suggesting pattern matching at phrase onsets based on rhythmical similarity. In the discussion of the grand average the most interesting feature is the negative shift in the time range 300–600 ms with a maximum in the fronto-central brain. This is ascribed to recognition of pattern similarity at phrase onsets with exactly the same rhythmical structure. The maximum amplitudes measured in the frontal parts of the brain suggest that non-expert listeners use the frontal part working memory for musical pattern recognition processes.

#### Magnetoencephalography (MEG)

**Figure 13:**
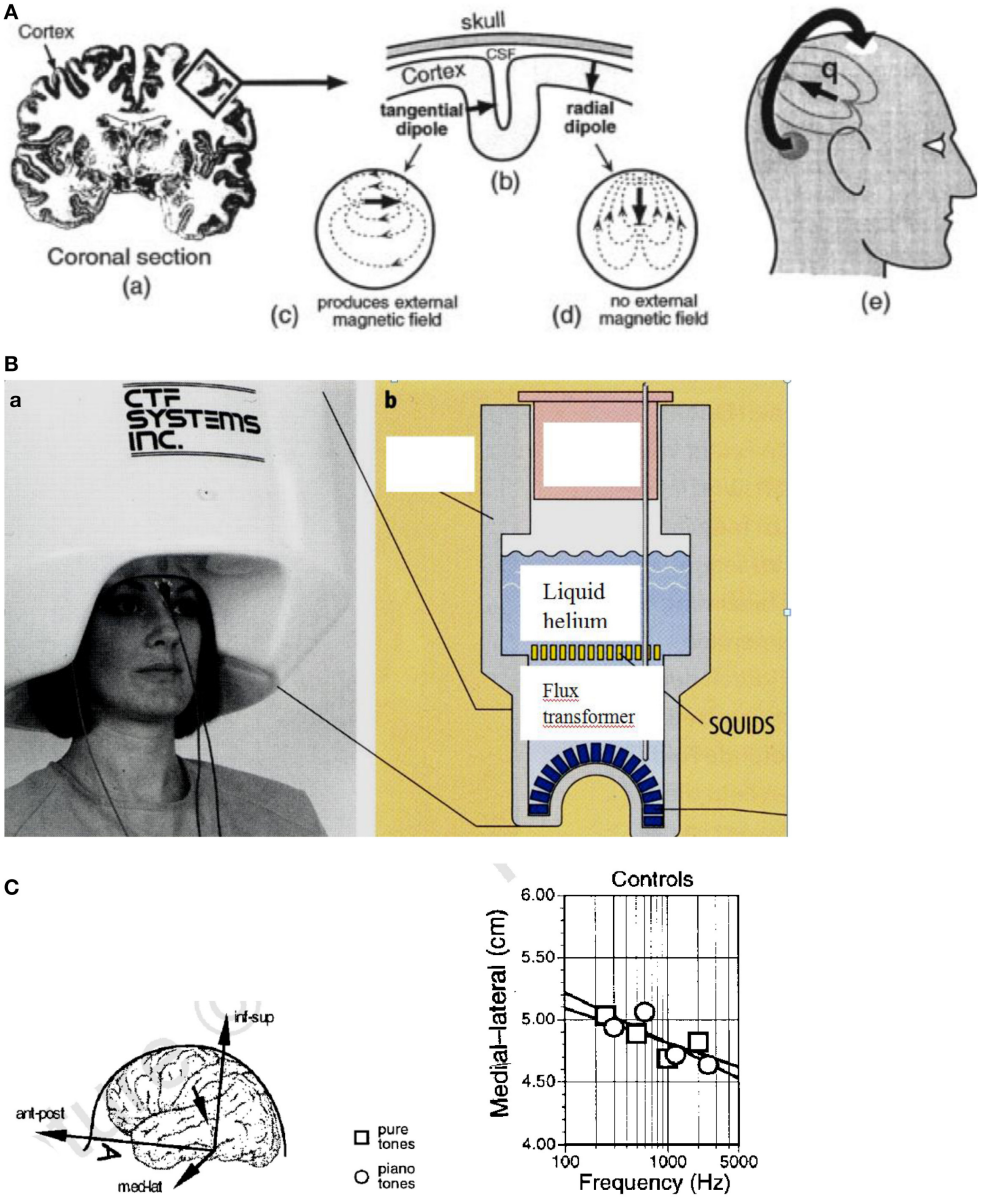
**(A)** Origin of the MEG signal. (a) Coronal section of the human brain with the cortex in dark color. The electrical currents flow roughly perpendicular to the cortex. (b) In the convoluted cortex with the sulci and gyri the currents flow either radially or tangentially (c) or radially (d) in the head. (e) The magnetic fields generated by the tangential currents can be detected outside the head (Vrba and Robinson, 2001). Reprinted with permission from Vrba and Robinson (2001) © 2001 Elsevier. **(B)** (a) Magnetoencephalography facility containing 150 magnetic field sensors. (b) SQUIDs (superconducting quantum interference devices) and sensors immersed for cooling in liquid helium contained in a Dewar vessel (cross section) (Birbaumer and Schmidt, 2010). Reprinted with permission from Birbaumer and Schmidt (2010) © 2010 Springer. **(C)** Cortical stimulation by pure and piano tones*. Left*: Medial–lateral coordinates are shown for single equivalent current dipoles fitted to the field patterns evoked by pure sine tones and piano tones in control subjects. The inset defines the coordinate system of the head. *Right*: Equivalent current dipoles (ECD) shift toward the sagittal midline along the medial–lateral coordinate as a function of the frequency of the tone. Ant–post, anterior–posterior; med–lat, medial–lateral; inf–sup, inferior–superior (Pantev et al., 1998). Reprinted with permission from Pantev et al. (1998) © 2001 Nature Publishing Group.

Weak magnetic fields which can be detected on the scalp are generated by the electrical currents in the brain (Figure 13A). By measuring these magnetic fields by a highly sensitive detector (Figure 13B), a tomographic image (MEG) of the brain activities can be reconstructed. The brain comprises about 2 × 10^10^ cells and about 10^14^ synapses. The dendritic current in the cell (see Figure 13A) generally flows perpendicular to the cortex (Figure 13A). In the case of the sulcus, this gives rise to a magnetic field in parallel to the scalp which is suggested to be detected outside when about 100,000 cells contribute, e.g., in the auditory cortex, with a spatial resolution of about 2–3 mm (Vrba and Robinson, 2001).

The brain magnetic fields (10^−13^ Tesla) are much smaller than the earth magnetic field (6.5 × 10^−5^ Tesla) and much smaller than the urban magnetic noise (10^−6^ Tesla) (Vrba and Robinson, 2001). The only detectors resolving these small fields are superconducting quantum interference devices (SQUIDs) based on the Josephson effect (see Figure 13B). The SQUIDs are coupled to the brain magnetic fields using combinations of superconducting coils called flux transformers (primary sensors, see Figure 13B).

One of the most successful methods for noise elimination is the use of synthetic higher-order gradiometers. A number of approaches is available for image reconstruction of the MEG signals. Present MEG systems incorporate several hundred sensors in a liquid helium helmet array (see Figure 13B).

By MEG scanning, neuronal activation in the brain can be monitored locally (Vrba and Robinson, 2001). Acoustic stimuli are processed in the auditory cortex by neurons that are aggregated into “tonotopic” maps according to their specific frequency tunings (see Pantev et al., 1998). In the auditory cortex, the tonotopic representation of the cortical sources corresponding to tones with different spectral content distributes along the medial-lateral axis of the supratemporal lane (see Figure 13C, left), with the medial-lateral center of the cortical activation shifting toward the sagittal midline with increasing frequency (see Figure 13C, right). This shift is less pronounced for a piano tone than for a pure sine tone. In this study, it could be additionally shown that dipole moments for piano tones are enhanced by about 25% in musicians compared with control subjects who had never played an instrument (Pantev et al., 1998). In the evaluation of the MEG data, for each evoked magnetic field a single equivalent current dipole (ECD) of about 50 nA was derived by a fit. From that a contribution of ~150,000 dendrites to this magnetic field can be estimated (Pantev et al., 1998). The coordinates of the dipole location were calculated satisfying the requirements of an anatomical distance of the ECD to the midsagittal plane of >2 cm and an inferior-superior value of >2 cm.

### Skin conductance response (SCR) and finger temperature

**Figure 14:**
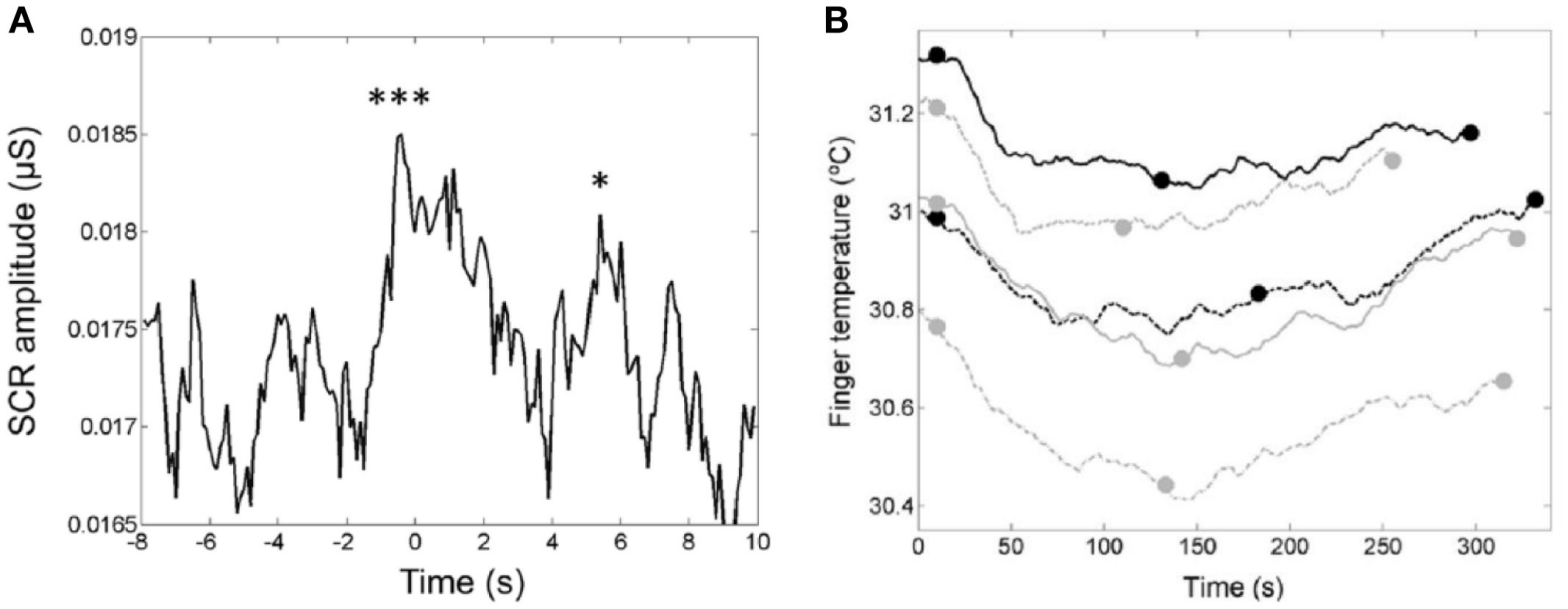
**(A)** The median curve of the skin conductance response (SCR) amplitude around the entrance of the chorus. The first downbeat was set to *t* = 0 s (Tsai et al., 2014). The two peaks are ascribed to the two closely related phases of listening experience: anticipatory “wanting” and hedonic “liking” of rewards. Reprinted with permission from Tsai et al. (2014) © 2014 Sage. **(B)** The u-shaped time-dependence of the finger temperatures of the listeners during presentation of the five songs. The end of the first chorus (see full dots) devides each song into two parts with a decrease of the finger temperature in the first part and an increase in the second part (Tsai et al., 2014). Reprinted with permission from Tsai et al. (2014) © 2014 Sage. The symbols ^***^ and ^*^ indicate that the two peaks are significantly larger than the control data.

In a study of the relationship of the temporal dynamics of emotion and the verse-chorus form of five popular “heartbreak” songs, the listeners' skin conductance responses (SCR; Figure 14A) and finger temperatures (Figure 14B) were used to infer levels of arousal and relaxation, respectively (Tsai et al., 2014). The passage preceding the chorus and the entrance of the chorus evoked two significant skin conductance responses (see Figure 14A). These two responses may reflect the arousal associated with the feelings of “wanting” and “liking,” respectively. Brain-imaging studies have shown that pleasurable music activates the listeners' reward system and serves as an abstract reward (Blood and Zatorre, 2001). The decrease of the finger temperature (Figure 14A) within the first part of the songs indicated negative emotions in the listeners, whereas the increases of the finger temperature within the second part may reflect a release of negative emotions. These findings may demonstrate the rewarding nature of the chorus and the cathartic effects associated with the verse-chorus form of heart-break songs.

### Goose bumps—piloerection

The most common psychological elicitors of piloerection or chills are moving music passages, or scenes in movies, plays, or books (see Benedek and Kaernbach, 2011). Other elicitors may be heroic or nostalgic moments, or physical contact with other persons. In Charles Darwin's seminal work on *The expression of emotions in Man and Animals* (1872), he already acknowledged that “…hardly any expressive movement is so general as the involuntary erection of the hairs…” (Darwin, 1872). Musical structures for triggering goose bumps or chills are considered to be crescendos, unexpected harmonies, or the entry of a solo voice, a choir, or a an additional instrument. It thus was concluded that piloerection may be a useful indicator which marks individual peaks in emotional arousal. Recently optical measuring techniques have been developed for monitoring and analyzing chills by means of piloerection (Benedek et al., 2010).

Additional experimental studies had shown that chills gave rise to higher skin conduction, increased heart and respiratory rates, and an enhancement of skin temperature (see Benedek and Kaernbach, 2011). Positron emission tomography correlated to musical chills showed a pattern typical for processes involved in reward, euphoria, and arousal, including ventral striatum, midbrain, amygdala, orbitofrontal cortex, and ventral medial prefrontal cortex (see Benedek and Kaernbach, 2011).

In the studies of piloerection as an objective and direct means of monitoring music-evoked emotion, music pieces ranging from 90 s (theme of *Pirates of the Caribbean*) to 300 s (*The Scientist*). Film audio tracks (*Knocking on Heavens Door, Dead Poets Society*) ranging from 141 to 148 s were employed. All musical stimuli were averaged to the same root mean square power (RMS), so that they featured equal average power.

**Figure 15:**
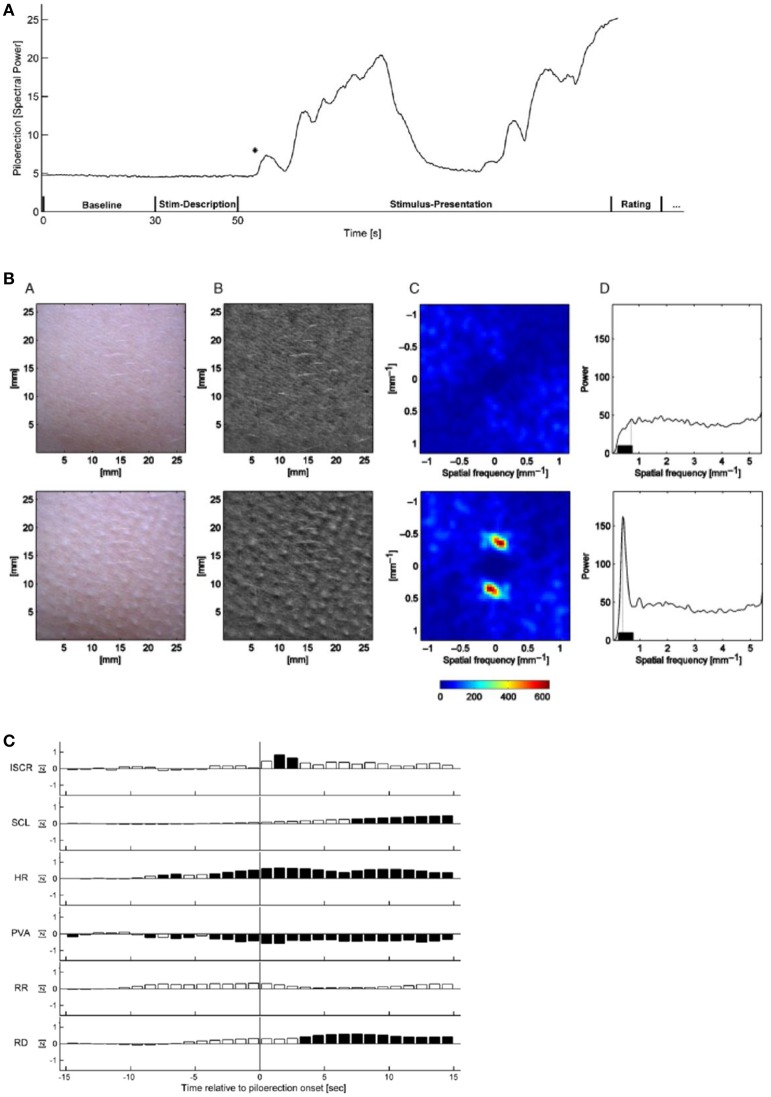
**(A)** Time-dependence of the relative piloerection intensity of a single experiment, including a baseline period (30 s), stimulus description (20 s) and stimulus presentation (variable duration). The initial stable level of piloerection intensity indicates no visible piloerection. In this experiment, piloerection occurs shortly after the onset of stimulus presentation; after some time it fades away. The asterisk marks the first detected onset of piloerection. This time is used for the short-term physiological response (Benedek and Kaernbach, 2011). Reprinted with permission from Benedek and Kaernbach (2011) © 2011 Elsevier. **(B)** Procedure of piloerection quantification without (top row) and with visible piloerection (bottom row). From B (bottom) a two-dimensional spatial Fourier transform is computed (C, shown for the frequency range ±1.13 mm^−1^) which is converted to a one-dimensional spectrum of spatial frequency. The maximum spectral power in the 0.23–0.75 mm^−1^ range (D) is considered as a correlate of the piloerection intensity (Benedek et al., 2010). Reprinted with permission from Benedek et al. (2010) © 2010 Wiley. **(C)** Time dependence of the short-term response of physiological measurements for a time slot of −15 s to +15 s around the first onset of piloerection. Dark bars indicate significant deviations from zero, white bars indicate non-significant deviations. ISCR-integrated skin conductance response, SCL-skin conductance level, HR-heart rate, PVA-pulse volume amplitude, RR-respiration rate, RD- respiration depth (Benedek and Kaernbach, 2011). Reprinted with permission from Benedek and Kaernbach (2011) © 2011 Elsevier.

Half of the musical stimuli (*My Heart will go on* by Celine Dion, *Only Time* by Enya, and film tracks of *Armageddon* and *Braveheart*) was pre-selected by the experimenter and half, with stronger stimulation, was self-selected by the 50 participants. The stimuli were presented via closed Beyerdynamic DT 770 PRO head-phones (Heilbronn, Germany) at an average sound pressure level of 63 dB. The procedure was approved by the Ethics Committee of the German Psychological Society (Benedek and Kaernbach, 2011). The sequence of a measurement is depicted in Figure 15A.

The formation of piloerection on the forearm was monitored by a video scanner with a sampling rate of 10 Hz, with simultaneous measurements of the skin conductance response and the increased heart and respiratory rates. By means of the Gooselab software the spatial Fourier transform (Figure 15B) of a video scan (Figure 15B) is derived which is a measure of the intensity of piloerection.

Piloerection could not always be detected objectively when indicated by the participant and was sometimes detected without an indication by the participant.

Piloerection starts with the onset of music (Figure 15A), then increases with a time constant of ~20 s and then fades off (time constant about 10 s). An analysis of the time constants of piloerection and of the kinetics of the simultaneously monitored physiological reactions (Figure 15C), should provide us with specific information on the neuronal and muscular processes contributing. This has not been discussed up to now. In the physiological quantities (Figure 15C) studied simultaneously with piloerection, a significant increase in skin conductance response, in heart rate, and in respiration depth has been observed. This demonstrates that a number of subsystems of the sympathetic neuronal system can be activated by music and that in particular listening to film sound tracks initiates a physiological state of intense arousal (Benedek and Kaernbach, 2011). Based on the experimental studies of piloerection and physiological quantities (Benedek and Kaernbach, 2011), two models of piloerection are discussed (Benedek and Kaernbach, 2011): On the one hand, it had been argued that the appearance of piloerection may mark a peak in emotional arousal (see Grewe et al., 2009). On the other hand, the psychobiological model (Panksepp, 1995) conceives emotional piloerection as an evolutionary relic of thermoregulatory response to an induced sensation of coldness and links it with the emotional quality of sadness (separation call hypothesis) (Panksepp, 1995). By comparing the physiological patterns of the two approaches to the experimental results, the authors (Benedek and Kaernbach, 2011) favor the separation call hypothesis (Panksepp, 1995) to the hypothesis of peak arousal (Grewe et al., 2009).

### Is there a biological background for the attractiveness of music?—genomic studies

In a recent genomic study, the correlation of the frequency of the listening to music and the availability of the arginine vasopressin receptor 1A (AVPR1A) gene or haplotype (with a length of 1,472 base pairs) has been investigated. A haplotype is a collection of particular d*eoxyribonucleic acid (DNA)* sequences in a cluster of tightly-linked genes on a chromosome that are likely to be inherited together. In this sense, a haplotype is a group of genes that a progeny inherits from one parent [http://en.wikipedia.org/wiki/Haplotype]. The AVPR1A gene encodes for a receptor molecule amino peptide that mediates the influence of the arginine vasopressin (AVP) hormone in the brain which plays an important role in memory and learning [http://en.wikipedia.org/wiki/Haplotype]. AVPR1A has been shown to modulate the social cognition and behavior, including social bonding and altruism in humans (Wallum et al., 2008). However, in contrast to that, the AVPR1A gene has also been referred to as the “ruthlessness gene” (Hopkin, 2008).

Recently an association of the AVPR1A gene with musical aptitude and with creativity in music, e.g., composing and arranging of music, has been reported (see Ukkola-Vuoti et al., 2011). In this study (Ukkola-Vuoti et al., 2011) a total of 31 Finnish families with 437 family members (mean age 43 years) participated. The musical aptitude of the individuals was tested by means of the Karma test. In this test, which does not depend on training in music, musical aptitude is defined as the ability of auditory structuring (Karma, 2007). In addition, the individual frequency of music listening was registered. Genomic DNA was extracted from peripheral blood of the individuals for the determination of the AVPR1A gene. The AVPR1A gene showed strongest association with current active music listening which is defined as attentive listening to music, including attending concerts. No dependence of the musical aptitude was discovered. These results appear to indicate a biological background for the attractiveness of music. The association with the AVPR1A gene suggests that listening to music is related to the neural pathways affecting attachment behavior and social communication (Ukkola-Vuoti et al., 2011).

## Towards a theory of musical emotions

In a recent overview (Juslin, 2013) aimed at a unified theory of musical emotions, a framework is suggested that tries to explain both the everyday emotions and aesthetic emotions, and yields some outlines for future research. This model comprises eight mechanisms for emotion by music—referred to as BRECVEMA: Brain stem reflexes, Rhythmic entrainment, Evaluative conditioning, Contagion, Visual imagery, Episodic memory, Musical expectancy, and Aesthetic judgment. The first seven mechanisms (BRECVEM) arousing the everyday emotions, are each correlated (see Juslin, 2013) to the evolutionary order, the survival value of the brain functions, the information focus, the mental representation, the key brain regions identified experimentally, the cultural impact, the ontogenetic development, the induced effect, the temporal focus of the effect, the induction speed, the degree of volitional influence, the availability of consciousness, and the dependence of musical structure.

Of particular significance is the addition of a mechanism corresponding to aesthetic judgments of music, in order to better account for typical appreciation emotions such as admiration and awe.

Aesthetic judgments have not received much attention in psychological research to date (Juslin, 2013) since aesthetic and stylistic norms and ideas change over time in society. Though it may be difficult to characterize aesthetic judgments, some preliminaries are offered (Juslin, 2013) as to how a psychological theory of aesthetic judgment in music experience might look like.

**Figure 16:**
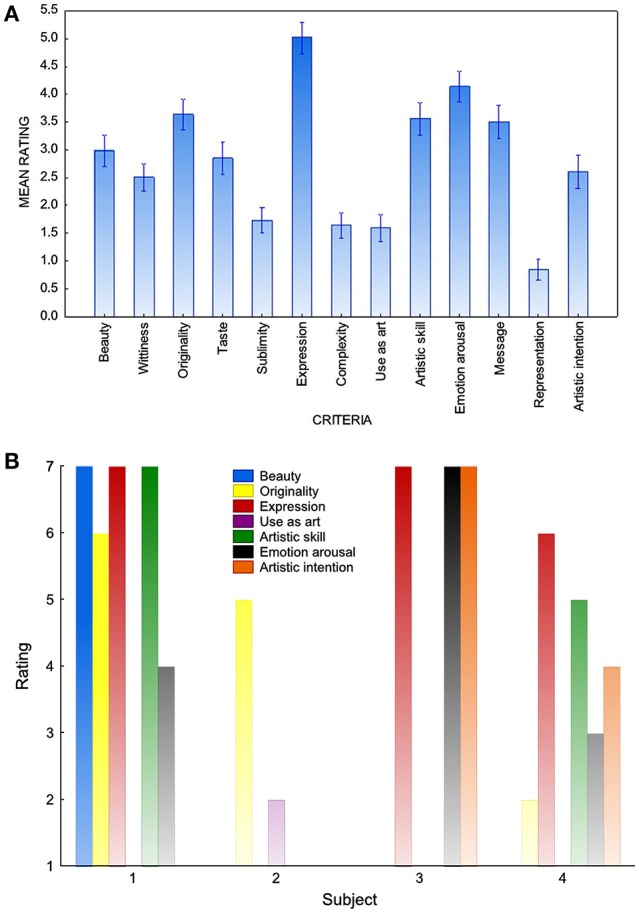
**(A)** Mean values and standard errors for listeners' ratings of criteria for aesthetic value of music. **(B)** Individual ratings of criteria for aesthetic value of music by four subjects (see Juslin, 2013). Reprinted with permission from Juslin (2013) © 2013 Elsevier.

Some pieces of music will invite an aesthetic attitude of the listener due to perceptual inputs by sensory impressions, due to more knowledge-based cognitive inputs, or due to emotional inputs. Some criteria that may underlie listeners' aesthetic judgments of music are suggested (Juslin, 2013) such as beauty, wittiness, originality, taste, sublimity, expression, complexity, use as art, artistic skill, emotion arousal, message, representation, and artistic intention. Certain criteria such as expression, emotional arousal, originality, skill, message, or beauty were considered as more important than others (see Figure 16A) and different listeners tend to focus on different criteria (see Figure 16B). With its multi-level framework of everyday emotions and aesthetic judgment, the study (Juslin, 2013) might help to explain the occurrence of mixed emotions such as bitter-sweet combinations of joy and melancholy.

This discussion suggests (Juslin, 2013) that researchers have to elaborate specific experimental paradigms that reliably arouse specific emotions in listeners through each of the mechanisms mentioned, including the empirical exploration of candidate-criteria for aesthetic value, similarly to what has been performed for various BRECVEM mechanisms. Empirical research so far has primarily focused on the beauty criterion (see Juslin, 2013). Developments of hypotheses for the criteria such as style appreciation, neural correlates of perceived expressivity in music performances, or perceptual correlates of novelty appear feasible (Juslin, 2013). An additional possibility could be the use of a neurochemical interference strategy (Chanda and Levitin, 2013; Juslin, 2013). It has been shown that blocking of a specific class of amino acid receptors in the amygdala can interfere with the acquisition of evaluative conditioning (see Juslin, 2013) discussed within BRECVEM. Interactions between BRECVEM mechanisms and aesthetic judgments have yet to be investigated.

## Musical therapy for psychiatric or neurologic impairments and deficiencies in music perception

Mounting evidence indicates that making music or listening to music activates a multitude of brain structures involved in cognitive, sensorimotor, and emotional processing (see Koelsch and Stegemann, 2012). The present knowledge on the neural correlates of music-evoked emotions and their health-related autonomic, endocrinological, and immunological effects could be used as a starting point for high-quality investigations of the beneficial effects of music on psychological and physiological health (Koelsch and Stegemann, 2012).

Music-evoked emotions can give rise to autonomic and endocrine responses as well as to motoric expression of motion (facial expression). The evidence that music improves health and well-being through the engagement of neurochemical systems for (i) reward, motivation and pleasure; (ii) stress and arousal; (iii) immunity; and (iv) social affiliation has been reviewed (Chanda and Levitin, 2013). From these observations, criteria for the potential use of music in therapy should be derived.

Dysfunctions and structural abnormalities in, e.g., the amygdala, hippocampus, thalamus, nucleus accumbens, caudate, and cingulate cortex are characteristic in pychiatric and neurological disorders, such as depression, anxiety, stress disorder, Parkinson's disease, schizophrenia, and neurodegenerative diseases. The findings that music can change the activity in these structures should encourage high-quality studies (see Koelsch, 2014) of the neural correlates of the therapeutic effects of music in order to provide convincing evidence for these effects (Drevets et al., 2008; Maratos et al., 2008; Omar et al., 2011). The activation of the amygdala and the hippocampal formation by musical chills as demonstrated in PET scans (Blood and Zatorre, 2001) may give direct support to the phenomenological efforts in music-therapeutic approaches for the treatment of disorders such as depression and anxiety because these disorders are partly ascribed to dysfunctions of the amygdala and presumably of the hippocampus (Koelsch and Stegemann, 2012).

Another condition in which music should have therapeutic effects is autism spectrum disorder (ASD). Functional MRI studies show (Caria et al., 2011) that individuals with ASD exhibit relatively intact perception and processing of music-evoked emotions despite their deficit in the ability to understand emotions in non-musical social communication (Lai et al., 2012). Active music therapy can be used to develop communication skills since music involves communication capabilities (Koelsch, 2014).

With regard to neurodegenerative disorders, some patients with Alzheimer's disease (AD) have almost preserved memory of musical information for, e.g., familiar or popular tunes. Learning of sung lyrics might lead to better retention of words in AD patients and anxiety levels of these patients can be reduced with the aid of music. Because of colocalization of memory functions and emotion in the hippocampus, future studies are suggested to more specifically investigate how music is preserved in AD patients and how it can ameliorate AD effects (Cuddy et al., 2012) and other neurodegenerative diseases such as Parkinson's disease (Nombela et al., 2013). In addition, music-therapeutical efforts for cancer (Archie et al., 2013) or stroke (Johansson, 2012) have been reported.

Music has been shown to be effective for the reduction of worries and anxiety (Koelsch and Stegemann, 2012) as well as for pain relief in clinical settings with, however, minor effects compared to analgesic drugs (see Koelsch, 2014). Deficiencies in music perception are reported for patients with cerebral degeneration or damage (Koelsch, 2014). Recognition of music expressing joy, sadness, anger, or fear is impaired in patients with frontotemporal lobar degeneration or damage of the amygdala (Koelsch, 2014). Patients with lesions in the hippocampus find dissonant music pleasant in contrast to healthy controls who find dissonance unpleasant. The degree of overlap between music-evoked emotions and so-called everyday emotions remains to be specified.

## Conclusions and outlook

As shown by tomographic imaging (fMRI, PET), which exhibits a high spatial resolution, activation of various brain areas can be initiated by musical stimuli. Some of these areas can be correlated to particular functions such as motor or auditive functions activated by non-musical stimuli. In the case of fMRI, emotion processing is identified by the more general feature of local energy consumption. Imaging of emotional processing on a molecular level can be achieved by PET, where specific molecules such as ^11^C-NMSP have been employed (Zhang et al., 2012) for a targeted investigation of synaptic activity (Zhang et al., 2012). A powerful combination of specific detection of molecules and tomographic imaging of the brain could arise from a future development of Raman tomography (Demers et al., 2012). Raman scattering provides specific information on the characteristic properties of molecules, such as vibrational or rotational modes.

Development of the technically demanding tomographic methods (fMRI, PET, MEG) for easy use would be highly desirable for the investigation of the emotions of performing musicians or even the astounding sensations of composers while composing, as, e.g., expressed by Ennio Morricone, composer of the music of the film *Once upon a time in the West* (Spiel mir das Lied vom Tod, 1968): “Vermutlich hat der Komponist, während er ein Stück schreibt, nicht mal die Kontrolle über seine eigenen Emotionen” (Morricone, 2014, Jun 1). (The composer, when witing a piece, is probably not even in control of his own emotions). Jörg Widmann, composer of the contemporary opera *Babylon* (2012), formulates: “Man gerät beim Schreiben in extreme Zustände, kann nicht schlafen, macht weiter in einer Art Rausch – und Rausch ist womöglich der klarste Zustand überhaupt.” (Widmann, 2014, August 20) (When composing one gets into extreme states, cannot sleep, continues in a sort of drunkenness—and drunkenness is perhaps the clearest possible state).

**Figure 17:**
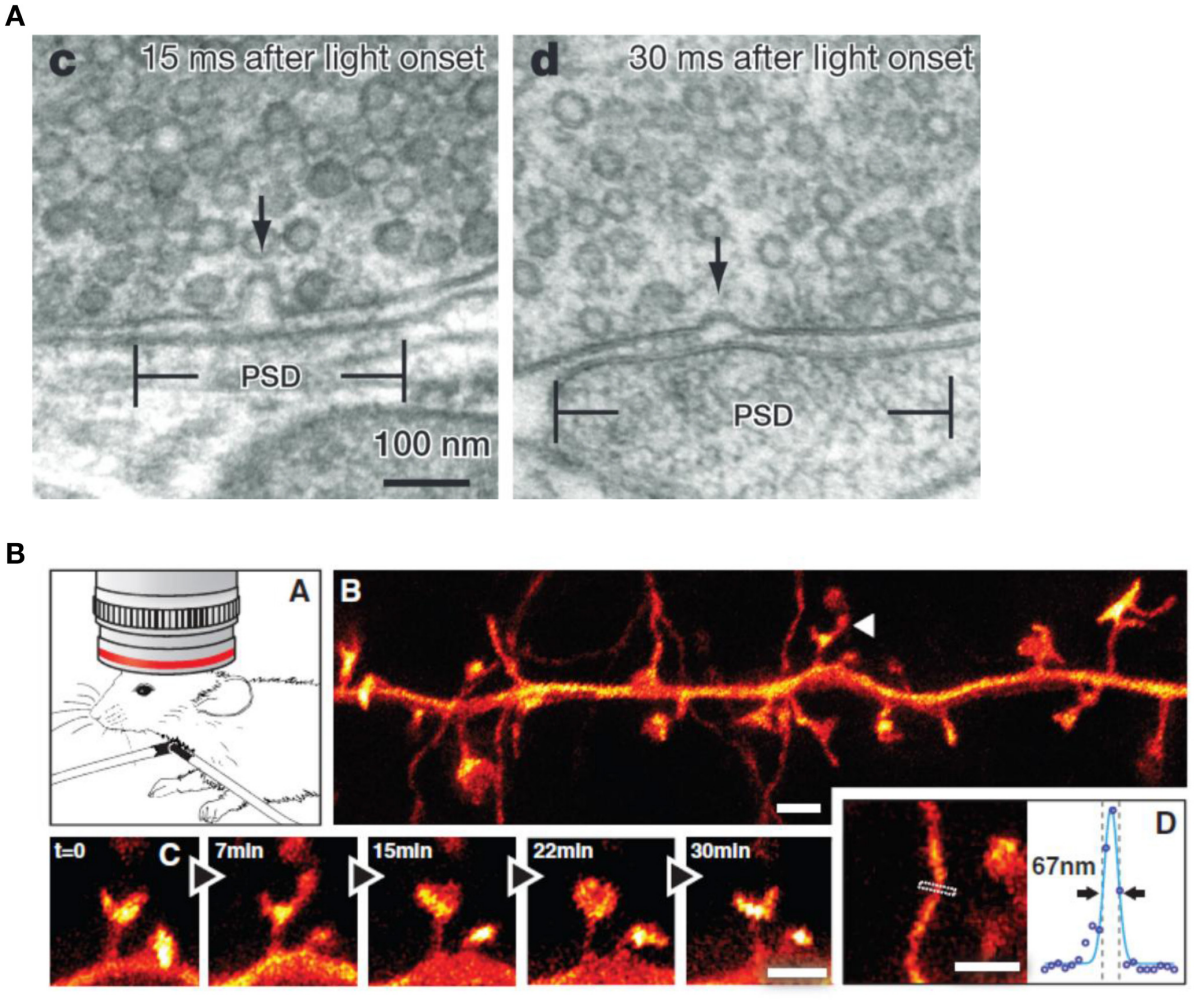
**(A)** Representative cryo electron micrographs of fusing vesicles (see arrows) in mouse hippocampal synapses at 15 ms (c) and 30 ms (d) after light onset (Watanabe et al., 2013). Reprinted with permission from Watanabe et al. (2013) © 2013 Nature Publishing Group. **(B)** STED (stimulated emission depletion) microscopy in the molecular layer of the somatosensory cortex of a mouse with EYFP-labeled neurons. (A) Anesthetized mouse under the objective lens. (B) Projected volumes of dendritic and axonal structures reveal (C) temporal dynamics of spine morphology with (D) an approximately four-fold improved spatial resolution compared with diffraction limited imaging. The curve is three-pixel-wide line profile fitted to raw data with a Gaussian. Scale bars, 1 μm (Berning et al., 2012). Reprinted with permission from Berning et al. (2012) © 2012 AAAS.

Future studies on a targeted molecular level may deepen the understanding of music-evoked emotion. Novel microscopy technologies for investigating single molecules are emerging. The rapid fusion of synaptic vesicles for neurotransmission after optical stimulation has been observed by cryo electron microscopy (Chemistry Nobel Prize 2017) with an electron energy of 200 keV where radiation damage appears tolerable and on a time scale of 15 ms (Watanabe et al., 2013) (see Figure 17A). Radiation damage can be entirely suppressed by combining electron holography and coherent electron diffraction imaging in a low- energy (50–250 eV) lens-less electron microscope with a spatial resolution of 0.2 nm (Latychevskaia et al., 2015). Of particular interest is the *in vivo* optical imaging of neurons (see Figure 17B) in the brain by STED (stimulated emission depletion) optical microscopy techniques (Chemistry Nobel Prize 2014) with a lateral resolution of 67 nm (Berning et al., 2012). The dynamics of the neuron spine morphology on a 7-min time scale (Figure 17B) potentially reflect alterations in the connectivity in the neural network characteristic for learning processes, even in the adult brain.

In addition, neurochemical interference strategies could be promising for future research as discussed in section Musical Therapy for Psychiatric or Neurologic Impairments and Deficiencies in Music Perception. For example, blocking of a specific class of amino acid receptors in the amygdala can interfere with the acquisition of evaluative conditioning (Juslin, 2013). In fact, studies of the neurochemistry of music may be the next great frontier (Chanda and Levitin, 2013), particularly as researchers try to investigate claims about the effects of music on health, where neurochemical studies are thought to be more appropriate than neuroanatomical studies (Chanda and Levitin, 2013).

The number of reports on beneficial effects of music on reward, motivation, pleasure, stress, arousal, immunity and social affiliation is mounting and the following issues could have future impact (Chanda and Levitin, 2013): (i) Rigorously matched control conditions in postoperative or chronic pain trials, including controls such as speeches, TV, comedy recordings etc. (ii) Experiments to uncover the neurochemical basis of pleasure and reward, such as through the use of the opioid antagonist naloxone in order to discover whether musical pleasure is subserved by the same chemical system as other forms of pleasure (Chanda and Levitin, 2013). (iii) Experiments to uncover the connection between oxytoxin (see Figure 11), group affiliation, and music (Chanda and Levitin, 2013). (iv) Investigation of the contribution of stress hormones, vasopressin, dopamine, and opioids in biological assays and pharmacological interventions together with neuroimaging (Chanda and Levitin, 2013).

The investigation of particular BRECVEM mechanisms (see section Musical Therapy for Psychiatric or Neurologic Impairments and Deficiencies in Music Perception) could be intensified through specific experiments. The interaction between BRECVEM mechanisms and aesthetic judgments has yet to be explored (Juslin, 2013). For an empirical exploration of candidate criteria for aesthetic judgment one has to map the characteristics of separate aesthetic criteria, as has been done with various BRECVEM mechanisms. Empirical research so far has focused on the beauty criterion (see Juslin, 2013) The more phenomenological measuring techniques such as encephalographic methods (EEG, MEG), skin conductance, and finger temperature or goose bump development characterized by a high time resolutions of 10 ms to 1 s are powerful tools for future observation of the dynamics and kinetics of emotional processing, where MEG can provide good time resolution together with moderate spatial resolution (Vrba and Robinson, 2001).

In addition to short-term studies, high-quality long-term studies would be desirable for the assessment of therapeutic efficacy over months in analogy to the year-long efforts of Carlo Farinelli for King Philipp V of Spain (see Section Historical Comments on the Impact of Music on People).

## Author contributions

H-ES selected the topic, performed the literature retrieval, and wrote the manuscript.

### Conflict of interest statement

The author declares that the research was conducted in the absence of any commercial or financial relationships that could be construed as a potential conflict of interest. The reviewer AF declared a shared affiliation, with no collaboration, with the author HS to the handling Editor.
