# Supplementary material for: Music-Evoked Emotions—Current Studies
Source: Front Neurosci. 2017 Nov 24;11:600. doi: 10.3389/fnins.2017.00600 (PMC5705548; doi:10.3389/fnins.2017.00600)
Supplement: Supplementary file 1 [file DataSheet1.docx]

## Appendix

**Psychological discussion of emotion**

Affective processes, states, traits, and pathways to their elucidation have been discussed earlier by psychologists (see refs.A1-A3), dating back to the early 20th century**A4**. In consideration of the necessity of an interdisciplinary approach at present, it becomes imperative to generate a minimal consensus about the defining features of the different types of affective phenomena.

Affective states with a number of design features (intensity, duration, synchronization etc.) were categorized**A1** into

- aesthetic emotions (admiration, ecstasy, fascination, harmony, solemnity),
- utilitarian emotions (anger, sadness, fear, joy, disgust, shame, guilt),
- preference ( like, dislike),
- mood (cheerful, gloomy, depressed, buoyant)
- interpersonal stances (distant, cold, warm)
- attitudes (loving, hating, desiring),and
- personality traits (nervous, anxious, morose, hostile, envious, jealous).

It has been suggested**A1** that none of the three major earlier assessment methods for emotion induction, namely

- discrete emotion theory**A3**, listing basic emotions, such as anger, fear, joy etc.,
- valence – activation two-dimensional space (valence: positive-negative, activation: arousal- calm), and
- eclectic emotion inventories (nostalgic, triumphant, exhilarating), is well suited to study emotional effects of music.

Given the large number of different affective states that can be induced by music, it has been thought necessary by psychologists**A1** to develop new methods of examining emotional verbal comments of music listeners in order to enable more objective assessments. Efforts have been

made**A1** to discover which verbal affect labels or terms, listeners to music of different types find most appropriate to label the affect state produced. By studying the effects of classical music, jazz, rock , and pop the appropriateness of about 10 terms for emotional verbal description is examined**A1**.

While the collection of the data of such a new scale might be feasible, the validation appears to be more of a problem. Criteria for this validation could be the agreement between different listeners to characterize similar pieces with similar scale terms, and the ability of the scale terms to discriminate different pieces of music. The characteristic terms could be selected according

to the frequency of their use and a minimum conceptual overlap. In the final list of terms, some terms may characterize the appraisal of intrinsic aesthetic quality. Others (like nostalgic or affectionate) suggest memory associations to places or relationships. Still others (such as comforted) may be linked to processes of empathy. The peripheral route consists of mechanisms which are based on motor induction of peripheral arousal. Terms like “feeling like dancing”, feeling energetic or strong but also calm suggest this type of mechanism.

It has been thought **A1** that a selection of this type of terms might provide more promising leads for

the inquiry into the underlying mechanisms of music-evoked emotions than established basic emotion lists or valence-activation dimensions.

In the interest of the comparability and cumulativeness of findings from different studies, it seems desirable to develop a standard list of emotion categories to be regularly employed in research using free response report of subjective feeling states and to use a reliable, standardized coding procedure. For this purpose, a modification of the two-dimensional representation of verbal assessments of emotion has been formulated**A2** by replacing the activation dimension by a high vs. low control/power (reflecting a coping potential appraisal check) in the so-called Geneva emotion wheel (see Fig. A1), including the representation of the intensity of the emotion as

the distance from the hub of the wheel and the size of the circles.

## References

A1. Scherer, K. R., (2004) Which emotions can be induced by music? What are the underlying mechanisms? And how can we measure them? *J. of New Music Research* **33**(3), 239-251.

A2. Scherer, K. R. (2005) What are emotions? And how can they be measured? *Trends and developments: research on emotion* **44**(4), 695-729.

A3. Ekman, P. (1992) An argument for basic emotions. *Cognition and Emotion,* ***6***, 169–200.

A4. Wundt, W. (1905) *Grundzüge der physiologischen Psychologie*. Leipzig: Engelmann.


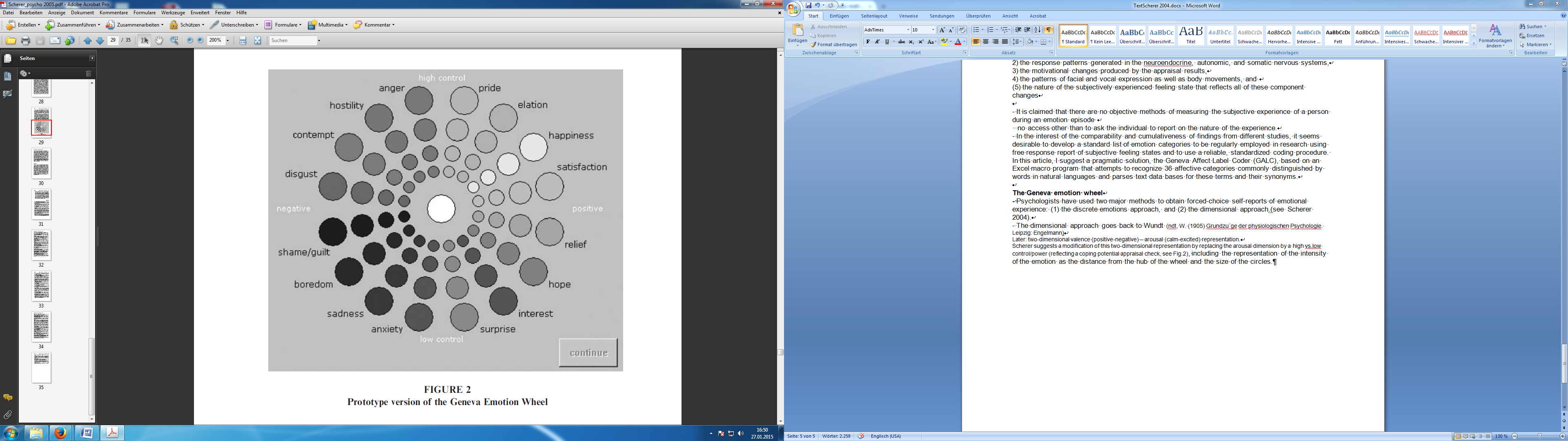


Fig. A1: Prototype version of the Geneva Emotion Wheel^A2^. Reprinted with permission from ref.
 A2 © 2005 Sage.
